# Pool size measurements facilitate the determination of fluxes at branching points in non-stationary metabolic flux analysis: the case of *Arabidopsis thaliana*

**DOI:** 10.3389/fpls.2015.00386

**Published:** 2015-06-02

**Authors:** Robert Heise, Alisdair R. Fernie, Mark Stitt, Zoran Nikoloski

**Affiliations:** ^1^Systems Biology and Mathematical Modeling Group, Max Planck Institute of Molecular Plant PhysiologyPotsdam, Germany; ^2^Central Metabolism Group, Max Planck Institute of Molecular Plant PhysiologyPotsdam, Germany; ^3^System Regulation Group, Max Planck Institute of Molecular Plant PhysiologyPotsdam, Germany

**Keywords:** flux profiling, *Arabidopsis thaliana*, metabolite pool sizes, metabolic flux analysis, photoautotrophic growth, isotopic labeling, isotopically non-stationary, carbon metabolism

## Abstract

Pool size measurements are important for the estimation of absolute intracellular fluxes in particular scenarios based on data from heavy carbon isotope experiments. Recently, steady-state fluxes estimates were obtained for central carbon metabolism in an intact illuminated rosette of *Arabidopsis thaliana* grown photoautotrophically (Szecowka et al., 2013; Heise et al., 2014). Fluxes were estimated therein by integrating mass-spectrometric data of the dynamics of the unlabeled metabolic fraction, data on metabolic pool sizes, partitioning of metabolic pools between cellular compartments and estimates of photosynthetically inactive pools, with a simplified model of plant central carbon metabolism. However, the fluxes were determined by treating the pool sizes as fixed parameters. Here we investigated whether and, if so, to what extent the treatment of pool sizes as parameters to be optimized in three scenarios may affect the flux estimates. The results are discussed in terms of benchmark values for canonical pathways and reactions, including starch and sucrose synthesis as well as the ribulose-1,5-bisphosphate carboxylation and oxygenation reactions. In addition, we discuss pathways emerging from a divergent branch point for which pool sizes are required for flux estimation, irrespective of the computational approach used for the simulation of the observable labeling pattern. Therefore, our findings indicate the necessity for development of techniques for accurate pool size measurements to improve the quality of flux estimates from non-stationary flux estimates in intact plant cells in the absence of alternative flux measurements.

## Introduction

Metabolism encompasses the entirety of largely enzyme-catalyzed reactions transforming the set of nutrients into molecules that support various functions. Metabolic reactions do not operate in isolation and collectively comprise functional networks, capable of bearing flux, fine-tuned by other levels of cellular organization (i.e., transcriptional regulation and signaling) (Stitt et al., 2010). Therefore, metabolic reaction fluxes are integrated outcomes of transcription and translation as well as their regulation. Reaction fluxes mutually relate to the involved metabolic pools and influence growth and other cellular tasks (Fell, 2005). For this reason, understanding the determinants of fluxes and their change upon perturbation is key to studying and controlling cellular behavior (Yoon et al., 2013). The determination of metabolic fluxes is challenging: fluxes cannot be measured directly and are, instead, estimated from measureable quantities. Classical approaches in plant physiology aim to infer the flux through a pathway from the accumulation of its end product (e.g., starch) (Sulpice et al., 2014) or by the accumulation of an applied tracer (e.g., ^14^C or ^13^C). Short-term radioactive labeling is also used to estimate the rate of synthesis of intermediates (Lunn and Hatch, 1995). Both approaches require the accumulation of the end product or the tracer within the time frame of the experiment. Here we refer to such approaches as *alternative flux measurements*. These approaches depend on a set of assumptions (e.g., that degradation does not happen concomitantly with accumulation in the case of an end product, Sweetlove et al., 1996) which should be critically examined before usage in a specific system. Nowadays, other more mathematically involved methods have been developed to facilitate the estimation of fluxes through selected components but also at a network level, as detailed below.

Based on the assumption of metabolic steady-state, two approaches have emerged to predict and estimate metabolic fluxes, namely, flux balance analysis (Williams et al., 2008, 2010; Orth et al., 2010; Sweetlove and Ratcliffe, 2011) and ^13^C metabolic flux analysis (^13^C-MFA) (Matsuoka and Shimizu, 2010; Zamboni, 2010; Niedenführ et al., 2014; Buescher et al., 2015), respectively. FBA predicts optimal flux distributions for a given objective based on a stoichiometric model. In contrast, ^13^C-MFA seeks data-driven support of the flux distribution based on ^13^C tracer studies in combination with a model. Heavy isotopes of carbon, e.g., ^13^C, have been widely used as tracers (label) to elucidate fluxomes, initially of prokaryotes, and recently of more complex eukaryotes including plants (Ratcliffe and Shachar-Hill, 2006; Allen et al., 2009; Kruger and Ratcliffe, 2009, 2015). Organisms grown on ^13^C-labeled substrates incorporate the label into metabolism, resulting in the appearance of different isotopomers of the involved metabolites. Although measurements of the complete isotopic composition of a metabolite pose challenges, labeling state of metabolites can be observed by either mass spectrometric (MS) (Antoniewicz, 2013) or nuclear magnetic resonance (NMR) (Fan and Lane, 2012) technologies. While MS allows resolving mass isotopomers of entire metabolites or of fragments of metabolites, NMR enables to measure positional labeling information. The observable labeling states represent sums of different isotopomers. A mathematical model is then used to describe these labeling states in their dependence to flux distributions, considered as free parameters fitted to the data. To this end, carbon transition maps (CTMs) are used to derive a mathematical model of isotopomer balance equations (Wiechert and De Graaf, 1997). CTMs are necessary to describe the generation of labeled products from their substrates in a cleavage-like reaction (Hörl et al., 2013).

First, we briefly outline the most prominent approaches in ^13^C-MFA, assuming that the system is in a metabolic steady state, and their applicability to estimate fluxes in plant cells. We then motivate and critically discuss the requirement and advantages of pool size measurements to estimate fluxes. The well-established method of isotopic stationary ^13^C-MFA (stat.-^13^C-MFA) describes isotopic stationary labeling pattern (Wiechert et al., 1997). The method allows resolving flux ratios of alternative pathways at merging points of a reaction network, if the merging fluxes contribute in distinguishable fashion to the observed labeling pattern at such points. Therefore, the applicability of the method depends strongly on the way the label is applied. Since the isotopic stationary labeling patterns are independent of the metabolic pool sizes, the method does not depend on pool sizes. The flux ratio at branch points in a metabolic network cannot always be estimated by stat.-^13^C-MFA. The ratio can only be determined if the branched pathways merge downstream the branch point. We refer to a branch point which does not merge downstream as a *divergent branch point*. To estimate fluxes at a divergent branch point one requires alternative flux measurements (see definition above). While stat.-^13^C-MFA has provided valuable insights into fluxes in prokaryotes grown on specifically labeled substrates (Wiechert, 2001) as well as plant cell cultures (Williams et al., 2008), its application to intact plants is hampered by several problems. These problems are due not only to the nature of the introduced label but also the complexity of plant cell (Allen et al., 2009; Kruger et al., 2012). While photoautotrophic growth offers an easy way to provide labeled carbon atoms as ^13^CO_2_ to the plant with a minimal disturbance, stat.-^13^C-MFA fails to resolve fluxes. In this case, all metabolic pools are fully labeled at isotopic steady-state, irrespectively of the fluxes operating in the system. Furthermore, flux ratios at branching points, which cannot be estimated by stat.-^13^C-MFA in absence of alternative flux measurements, become of interest, especially in the case of secondary metabolism. In addition, ^13^C-MFA in plants is hampered by the compartmentalization and parallel pathways at the level of tissue and cell (Ratcliffe and Shachar-Hill, 2006; Allen et al., 2009). This is largely due to the difficulty of measuring compartment-specific labeling states, since existing methods allow the quantification of the sum of all pools of a metabolite in the sample. Moreover, compartmentalized metabolic pools can take part in different biochemical pathways and transport reactions have to be taken into consideration.

The introduction of isotopic non-stationary ^13^C-MFA (non-stat.-^13^C –MFA, also called isotopic instationary ^13^C-MFA) has aimed at overcoming these problems by modeling the dynamic toward isotopic steady state (Shastri and Morgan, 2007; Nöh and Wiechert, 2011), and several publication already discuss the benefits of non-stationary ^13^C-MFA in greater detail (Nöh and Wiechert, 2011; Wiechert and Nöh, 2013). The approach requires the monitoring of the labeling process at a sufficient number of time-points and necessitates rapid quenching of the samples. The mathematical description of the process comprises a system of ordinary differential equations (ODEs), whose solution gives the time-course of the labeling states of the involved metabolic pools. The mathematical system of equations, and therefore its solution, depends on the distribution of the metabolic flux and, in contrast to stat.-^13^C -MFA, on the metabolic pool sizes (Nöh and Wiechert, 2011). The system of differential equations incorporates information about the carbon transition in the metabolic network. In the following we describe the general structure of the system of ODEs capturing the dynamic of the isotopomers in a isotopomer network. Such a system is complete in the sense that its solution, given by the time-course of all isotopomers, allows calculating the time-course of an observable labeling pattern, such as mass isotopomers.

The dynamics of the absolute abundance *x_m, i_* of an isotopomer *i* in the metabolic pool *m* can be described by the following ODE:

$$\frac{dx_{m,i}}{dt}=\sum_{r}F_{r,m}^{in}h_{r,m,i}\left(t\right)-\sum_{s}F_{s,m,i}^{out}\frac{x_{m,i}}{p_{m}},$$

where *F^in^_r, m_* denotes the metabolic steady-state flux of a reaction *r* in which pool *m* participates as a product, and *F^out^_s, m_* stands for the metabolic steady-state flux of a reaction *s* in which pool *m* participates as a substrate. The size of the metabolic pool *m* is denoted by *p_m_* and is given by the sum over the abundance of all isotopomers:

$$p_{m}=\sum_{i}x_{m,i}.$$

The function *h_r, m, i_*(*t*), with the interval [0, 1] as its range, describes the relative amount of newly synthesized molecules of isotopomer *i* in pool m via *F^in^_r, m_*. It depends on the relative amount of the isotopomers of all substrates of reaction *r* which generate the isotopomer denoted by, i.e.,

$$h_{r,m,i}\left(t\right)=\prod_{n}\sum_{j\in S_{n,r,m,i}}\frac{x_{n,j}(t)}{p_{n}}.$$

The absolute abundance of a particular isotopomer of a substrate pool *n* resulting in the generation of *x_m, i_* is denoted by *x_n, j_*. The set of indices of all such isotopomers is denoted by *S_n, r, m, i_* (hence, all *h_r_*(·) encode information about CTMs via the respective sets of indices in *S_n, r, m, i_*). Since each molecule in the pool has the same probability to react, the relative amount of further reacting molecules of isotopomers *i* via *F^out^_s_* is given by the relative amount of *i* in the pool *m*. Furthermore, if the system is in a metabolic steady state, the sum of all *F^in^_r, m_* equals the sum of all *F^out^_s, m_* as well as the total flux through the pool *m*, denoted by *F_m_*. The ODE above can then be written as:

$$\frac{dx_{m,i}}{dt}=\sum_{r}F_{r,m}^{in}h_{r,m,i}\left(t\right)-\frac{F_{m}}{p_{m}}x_{m,i}.$$

This ODE can be applied to isotopomer reaction network resulting in a system of ODEs, in which there is an ODE for each isotopomer. The solution of the system describes the time-course of the absolute isotopomer abundance and can be fitted to the experimentally determined data. Therefore, while such a procedure enables the estimation of absolute metabolic fluxes and pool sizes, it implicitly demands the quantification of the absolute isotopomer abundance (and, thereby, absolute metabolic pool sizes).

Alternatively, the fractional contribution $\tilde{x}$*_m, i_* of the isotopomers can be measured, which may be more easily accessible. The ODE can then be scaled to describe the fractional contribution of the isotopomer *i* to its pool *m* as:

$$\frac{d{\tilde{x}}_{m,i}}{dt}=\frac{F_{m}}{p_{m}}\sum_{r}\frac{F_{r,m}^{in}}{F_{m}}h_{r,m,i}\left(t\right)-\frac{F_{m}}{p_{m}}{\tilde{x}}_{m,i}\left(t\right),$$

where ${\tilde{x}}_{m,\;i}(t)\;=\;\frac{x_{m,\;i}}{p_{m}}$. This ODE depends on *h_r, m, i_*(*t*), the flux ratios, $\alpha_{r,\;m}\;=\;\frac{F_{r,\;m}^{in}}{F_{m}}$, and the total-flux-to-pool-size ratio, $k_{m}=\frac{F_{m}}{p_{m}}$. The relative amount of newly synthesized isotopomers *i* of metabolite *m* via all reactions, can be summarized by the effective relative generation of $\tilde{x}$*_m, i_*, given by:

$$h_{m,i}^{eff}\left(t\right)=\sum_{r}\frac{F_{r,m}^{in}}{F_{m}}h_{r,m,i}\left(t\right).$$

This leads to the following inhomogeneous first-order differential equation:

$$\frac{d{\tilde{x}}_{m,i}}{dt}=k_{m}h_{m,i}^{eff}-k_{m}{\tilde{x}}_{m,i}$$

whose solution can be expressed as:

$${\tilde{x}}_{m,i}\left(t\right)=k_{m}\int_{-\infty }^{t}h_{m,i}^{eff}\left(t-\tau \right)e^{-k_{m}\tau }d\tau ,$$

with *h^eff^_m, i_*(*t*) = $\tilde{x}$*_m, i_* (0) for *t* < 0.

We note that the ratio *k_m_* acts as a time constant which affects the relaxation of $\tilde{x}$*_m, i_*(*t*) toward *h^eff^_m, i_*(*t*), resulting in a delay between their time courses (trajectories). A large time constant, caused by a high total flux *F_m_* or a low pool size *p_m_*, results in a small delay and $\tilde{x}$*_m, i_*(*t*) is dominated by *h^eff^_m, i_*(*t*), i.e.,

$$\underset{k_{m}\to \infty }{\lim}{\tilde{x}}_{m,i}(t)=h_{m,i}^{eff}\left(t\right)=\sum_{r}\frac{F_{r,m}^{in}}{F_{m}}h_{r,m,i}\left(t\right).$$

Fitting the solution for $\tilde{x}$*_m, i_*(*t*) to time-resolved data of the fractional contribution of an observable labeling pattern allows the estimation of flux ratios, α*_r, m_*, at merging points of a reaction network and the specific time constant, *k_m_*, of the pool *m*. We would like to note that both the flux ratio and the time constant are characteristic for the metabolic pool *m*, since the ODEs for all isotopomers of the same metabolic pool are of the same values. Therefore, usage of additional data that describe the time-course of the labeling process of a metabolic pool, e.g., other mass isotopomers, typically results in a better determination of the flux ratios α*_r, m_* and the time-constant *k_m_*. Nevertheless, the flux ratios at a merging point can only be estimated if the contributions, *h_r, m, i_*(*t*), of different reactions to the composition of *h^eff^_m, i_*(*t*), is not identical for all reactions. The latter can even be possible in the case in which the contribution in isotopic steady state is indistinguishable, and stat.-^13^C-MFA will fail to resolve flux ratios at the merging point (e.g., like in photoautotrophic growth). Therefore, the possibility to address this issue represents a major advantage of non-stat.-^13^C-MFA. In the particular case when the time constant of the product pool is sufficiently high, and hence, it does not contribute to the time course of $\tilde{x}$*_m, i_*(*t*), the pool size, *p_m_*, does not have to be provided to estimate the flux ratios α*_r, m_*.

Another advantage of non-stat.-^13^C-MFA, as evident from the provided mathematical formulation, is the determination of the time constants, *k_m_*, which enables the calculation of absolute flux values *F_m_* given the measured pool size (since $k_{m}=\frac{F_{m}}{p_{m}}$). This principle allows the estimation of fluxes in single reactions as well as in linear chains and at branching points of a reaction network. We note that at each branching point, a single time constant is resolved for each reaction. Since the flux, *F_m_*, of a reaction is given by *F_m_* = *k_m_p_m_*, the ratio of the branching fluxes still depends on the ratio of the pool sizes. Furthermore, we note that flux ratios at branching points can also be determined at a global level if the branching pathways merge downstream of the branching point. However, if the number of branching points exceeds the number of merging points in a modeled network (with external in- and out- fluxes), the flux ratio cannot be determined. In this case, additional assumptions or measurements are needed to constrain the steady-state flux distribution (see further details below for existing applications).

Several approaches were developed to apply the principles of non-stat.-^13^C-MFA, described above. Kinetic Flux Profiling (KFP) (Yuan et al., 2006, 2008) resolves the time constant from the time-course of the unlabeled metabolic fractions, and can be used to estimate forward fluxes for single monomolecular reactions. Non-stationary flux ratio analysis (NFA) (Hörl et al., 2013) extends the idea to general reactions by usage of CTMs and allows the estimation of the flux ratios of influxes (at merging points). In addition, it incorporates all massisotopic fractions. As local approaches, both methods estimate the time course of generation of mass isotopomers by additional assumptions based on the time course of the labeling of the substrates. Local flux estimates for the forward fluxes of different reactions in a network can be employed to further analyze the entire flux estimates.

In contrast to these local methods, the dynamic of all (mass) isotopomers of all metabolites can be simulated in a global manner from the point of the application of the label. In this case, the mathematical model comprises a system of ODEs which incorporate knowledge of the biochemical pathways including the CTMs. While global non-stat.-^13^C-MFA is computationally intensive, for MS measurements the computational demands can be reduced by decomposing the network in its elementary metabolite units (EMUs) (Antoniewicz et al., 2007). The necessity of measurement of pool sizes in non-stat.-^13^C-MFA in the global approach has been questioned, since they can be directly included as parameters in the optimization (Wiechert and Nöh, 2005; Shastri and Morgan, 2007). The latter has been supported by the observation that pool size measurements may be more corrupted by losses during extraction and quenching in comparison to the labeling states (Wahl et al., 2008). Furthermore, metabolic pools might be in fast exchange with other pools, such that the combined, and not the individual, pool affects the observed time constant (Young et al., 2011). Since the effects of pool sizes on the observed time constant may even be more complicated, the corresponding estimated pool size might not reflect the measured pool size. Non-stat.-^13^C-MFA based on EMU decomposition has been applied to the cyanobacterium *Synechocystis* sp. PCC6803 photoautotrophically grown on ^13^CO_2_ (Young et al., 2011). Flux ratios and time constants were estimated and absolute fluxes as well metabolic pool sizes were calculated from the measured CO_2_ uptake rate.

However, also in the global modeling approach, fluxes or flux ratios of reactions on branched chains which do not merge cannot be estimated from the time courses of the (mass) isotopomers without additional knowledge of the pool sizes. A recent study applied global non-stat.-^13^C-MFA based on EMU decomposition to time-resolved labeling data of Arabidopsis rosettes grown on ^13^CO_2_ (Ma et al., 2014). The study estimates flux ratios and pool sizes. In particular, the ratio of the photorespiratory flux and the net (gross) carbon fixation were estimated. However, fluxes or flux ratios on branching chains were provided by independent measurements or constrained by additional assumptions. The flux of the synthesis of starch was estimated by the average starch accumulation and the ratio of fluxes of the synthesis of sucrose and amino acids was constrained by observed ratios of pool sizes.

The compartmentation of plant cells presents an additional problem in MFA. To this end, the measurement of compartment-specific labeling pattern is challenging. Alternatively, simulated time-courses of two pools $\tilde{x}$_1_(*t*) and $\tilde{x}$_2_(*t*) of the same metabolites can be weighted-summed to describe the measured time-course of the fractional metabolic content $\tilde{z}$(*t*) of the quantity expressed by $\tilde{x}$:

$$\tilde{z}\left(t\right)=\text{β}{\tilde{x}}_{1}\left(t\right)+\left(1-\text{β}\right){\tilde{x}}_{2}\left(t\right).$$

Additionally a metabolically inactive fraction φ can contribute to the measured content, the fractional metabolic content is given by:

$$\tilde{z}\left(t\right)=\frac{\text{β}{\tilde{x}}_{1}\left(t\right)+\left(1-\text{β}\right){\tilde{x}}_{2}\left(t\right)}{1-\text{φ}}+\text{φ}.$$

The metabolically inactive fraction corresponds to a metabolically inactive pool, which can occur due to compartmentation at the level of cell or tissue. Here, we use the notion of content to denote the sum of all pools of the same metabolite. The weight β can be an independent parameter or reflect the ratio of the pool sizes of the compartmented metabolite. Given the abovementioned arguments, the latter might be misleading in the case where the time constants are influenced by a combined pool of metabolites in rapid equilibrium. On the other hand, if this is not the case, it offers the additional possibility to resolve ratios of pool size and, thus, flux ratio in parallel pathways via inst.-^13^C-MFA (provided the involved compartmented metabolite label on distinguishable time scales). We would like to stress that the weight β can be determined experimentally, e.g., by non-aqueous fractionation (n.a.f.) (Gerhardt and Heldt, 1984; Stitt et al., 1989), while the estimation of the metabolically inactive fraction requires further assumptions and may pose further challenges (see Materials and Methods).

We recently published estimates of photosynthetic carbon fluxes in Arabidopsis rosettes based pool sizes as parameters to be optimized affect the correspondence between benchmark values for fluxes of canonical pathways. To this end, we included the variance-weighted differences between measured and estimated pool sizes to the variance-weighted sum of squared errors. Accordingly, we distinguished between time-course-related (referred to as time-related) and pool size error. We considered the scenarios in which the estimated pool sizes are either required to fall in the intervals of measured values or are practically unbounded. We used this procedure and scenarios to determine which metabolic pools, when optimized as free parameters, differ most from the measured quantities employed in the earlier studies to obtain absolute flux estimates. In addition, we conducted a sensitivity analysis which revealed the metabolic pools with large effect on the flux estimates and the variance-weighted difference due to the time course of the unlabeled fractions. Altogether, our study points at the necessity for accurate pool size measurements for reliable flux estimates in intact plant cells in the absence of alternative flux measurements.

## Materials and methods

### Plant material

The experimental procedure was performed as described in detail (Heise et al., 2014). Plants were grown in short 8-h day/16-h night cycles under an average irradiance of 115 μmol/m^2^/s. The temperature was 22°C day/20°C night and the relative humidity was 50%. Five-week-old plants were rapidly transferred from a growth to a labeling chamber, which was continuously washed through the whole experiment by a stream of air containing N_2_, O_2_, and ^13^CO_2_. Beginning with the transfer, the plants start fixing ^13^C. Plants were harvested 5 and 10 s and 1, 3, 10, 20, and 60 min after transfer. Samples were analyzed using three analytical platforms, namely, GC-TOF-MS, ion exchange LC-MS/MS and reverse-phase LC-MS/MS. The obtained data of the time-course of the unlabeled fraction was corrected for natural abundance of ^13^C.

### Flux analysis

The flux analysis was performed as described in detail in (Heise et al., 2014). The mathematical approach is based on simultaneous modeling of the time course of *all* unlabeled metabolic fractions at metabolic steady state and coincides with the simulation of EMU-state-variable of mass-state zero (Antoniewicz et al., 2007). For this purpose a system of ODEs is constructed from the considered pathway model. The pathway model describes photosynthetic active tissue and neglects effects related to growth or oxidative respiration. It comprises the fluxes through the Calvin–Benson cycle (CBC), a simplified photorespiratory pathway and the reactions involved in synthesis of starch, sucrose and trehalose (see Supplementary Figure 1). The steady-state flux distribution is described by 11 parameters ϕ as a linear combination of the corresponding 11 flux modes *M*:

$$F_{r}=\sum_{i=1}^{11}\phi^{i}M_{r}^{i}.$$

Four of these modes depict elementary flux modes (Supplementary Table 1), which describe the net flux distribution and considering all reactions of the CBC. The remaining 7 modes represent (futile) cycles, which describe the exchange flux (Wiechert and De Graaf, 1997; Wiechert, 2007), for each reversible reaction as the difference between forward and net flux or the backward flux, respectively. To avoid the computational demanding simulation of all intermediates of the CBC by three assumptions: (1) the time-course of the unlabeled fractions only depends on the labeling state of ribulose-1,5-bisphosphate (RuBP). All further influences of intermediates of the CBC are neglected. Therefore, all dependencies of the unlabeled fractions can be exclusively traced to RuBP (EMUs of RuBP) or CO_2_; (2) the time-course of the unlabeled fraction of CO_2_ is assumed to be zero from the beginning; and (3) a uniform distribution of label within each mass isotopomer of RuBP is assumed. The latter implies that each isotopomer of a particular mass isotopomer has the same relative abundance. As a consequence, all EMU-state-variables of RuBP of the same EMU-size are equal and can be simulated by a single variable. Therefore, our approach bridges the local and global approaches presented in the introduction, above. We note that an EMU-state-variable of mass-state zero coincides with a cumomer (Wiechert and De Graaf, 1997) fraction describing the sum of all isotopomers unlabeled at a particular position. The weight of a cumomer equals the size of the corresponding EMU and denotes the number of unlabeled positions. Following the assumption (3), the relative contribution θ*^i^_s_* of the *i*-th mass isotopomer to an unlabeled cumomer of size *s* is, thus, independent of the time and approximated by:

$$\theta_{s}^{i}\approx \frac{\left(\begin{array}{c} N-s\\ i \end{array}\right)}{\left(\begin{array}{c} N\\ i \end{array}\right)}.$$

This allows the description of the time-course of a cumomer fraction *y_RuBP, s_*(*t*) of RuBP and size *s* from the time-course of the mass isotopomer fraction $\tilde{x}$*^i^_RuBP_*(*t*) of RuBP:

$$y_{RuBP,s}\left(t\right)\approx \sum_{i=0}^{5}{\text{θ}}_{s}^{i}{\tilde{x}}_{RuBP}^{i}\left(t\right),$$

and relates the cumomer fraction *y_RuBP, s_*(*t*) with the measured massisotopic abundance $\tilde{x}$*^i^_RuBP_*(*t*) at a particular time point *t*. To model the time courses of the necessary cumomers fractions *y_RuBP, s_*(*t*), a sum of two exponential functions is fitted to available *y_RuBP, s_*(*t*) for each *s*:

$$y_{RuBP,s}^{input}\left(t\right)=A_{s}e^{-a_{s}t}+B_{s}e^{-b_{s}t},\text{ where }A_{s}+B_{s}=1.$$

We call these functions input models *y^input^_RuBP, s_*(*t*), whose parameters we fit prior to integrating the system of ODEs (Supplementary Table 2). The input models represent the inhomogeneous terms in the system of ODEs, provided in Supplementary Equations.

The experimentally obtained time courses of the most unlabeled metabolic fractions do not decay completely; instead, they plateau at a certain level. In addition, for some metabolites, this plateau is higher than the one of the direct downstream products (Szecowka et al., 2013). Therefore, the model assumes a metabolically inactive pool for all metabolites *m* with such behavior, expressed as the inactive fraction φ*_m_*. Such an inactive pool could result from the presence of several cell and tissue types in the Arabidopsis rosette which might not labeled within the time-frame of the experiment. For trehalose, the inactive fraction was fixed to zero, since a plateau was not observed.

Here, for a given metabolite, we distinguish between its pool size, which refers to the size of a particular modeled pool (e.g., in different compartments) and the metabolic content, which represents sum of all pools of the same metabolite. Compartmentalization data based on n.a.f. (Gerhardt and Heldt, 1984; Stitt et al., 1989) are used to distinguish between metabolites in different compartments. The term compartmentalized content *c_n_* refers either to the content of a metabolite for non-compartmented metabolites or to the content of a metabolite in a particular compartment, which was obtain from the n.a.f. data. For each pool *n* of the model a compartmentalized content *c_n_* is provided, which is factored in active and inactive pool sizes. The inactive fractions of two pools of the same metabolite in different compartments are assumed to be equal, since it is unclear how an inactive pool is partitioned based on the n.a.f. data.

The active pool sizes (obtained from measurements or estimated as free parameters) are used together with the system of ODEs to numerically integrate it; this results in the simulated time course of the unlabeled fraction $\tilde{x}$*_t, n_* of each pool *n*. The simulated $\tilde{x}$*_t, n_* were used to calculate the unlabeled fractional content $\tilde{z}$*_t, m_* of metabolite *m*. For the metabolites which appear with only one pool *n* (not partitioned between compartments), it is given by:

$${\tilde{z}}_{t,m}=\left(1-\varphi_{m}\right){\tilde{x}}_{t,n}+\varphi_{m},$$

while for the metabolites which appear with two pools, *n* and *l*, is given by:

$${\tilde{z}}_{t,m}=\left(1-\varphi_{m}\right)\frac{{\tilde{x}}_{t,n}p_{k}+{\tilde{x}}_{t,l}p_{l}}{p_{n}+p_{l}}+\varphi_{m}.$$

To obtain estimates of flux and/or pool size the variance-weighted sums of squares (*VWSS*) between was minimized. Here we distinguish between the error of time-course of the unlabeled metabolic content:

$$VWSS_{t}=\sum_{m}\sum_{t}\frac{{\left({\tilde{z}}_{obs.,t,m}-{\tilde{z}}_{sim.,t,m}\right)}^{2}}{\sigma_{{\tilde{z}}_{obs.,t,m}}^{2}},$$

and the error of the total metabolic content:

$$VWSS_{c}=\sum_{n}\frac{{\left(c_{obs.,n}-c_{sim.,n}\right)}^{2}}{\sigma_{c_{obs.,n}}^{2}}.$$

The observed and simulated unlabeled fractional content at time *t* of metabolite *m* was denoted as $\tilde{z}$*_obs., t, m_* and $\tilde{z}$*_sim., t, m_* and the metabolic content as *c_obs., n_* and *c_sim., n_*. For the analysis of different scenarios we use either *VWSS_t_* or the total error:

$$VWSS_{all}=VWSS_{t}+VWSS_{c},$$

The corresponding variance of observed values is denoted by σ^2^. To avoid over-fitting to single data points of the time course of measured unlabeled content, we substituted the variance of the data at each time point with the mean variance of the corresponding metabolite over all time points.

The numerical integration of the system of differential equation (see Supplementary Equations) is performed by the backward differentiation formula of sundials CVODE (Cohen and Hindmarsh, 1996) suitable for the integration of stiff systems. The optimization was performed by the Subplex algorithm (Rowan, 1990) suitable for local optimization. To avoid the possibility of local minima, we performed 100 repetitions and selected the best performing with respect to the error function. The initial conditions for the metabolic content were randomly chosen from assumed distributions (see Supplementary Table 3). The upper and lower boundaries of the optimization for the metabolic content are shown in Supplementary Table 3. The parameters describing the magnitude of an exchange flux were hyperbolically transformed (see Supplementary Table 3) (Wiechert and De Graaf, 1997). The confidence intervals were calculated by Monte Carlo simulation (Press et al., 2007) as described in (Heise et al., 2014).

### Sensitivity analysis

Each optimized parameter, corresponding to a particular metabolic content, was changed by 50, 60, 70, 80, 90, 95, 99, 101, 105, 110, 120, 130, 140, 150% from its value in the optimum. The system was re-optimized while keeping the selected content fixed to the imposed relative change. A linear regression was then fitted for the relative change in the parameter whose value was kept fixed and the relative change in the re-optimized quantity of interest (fluxes and time-related error). The slope of the obtained line could be regarded to express the sensitivity of the quantity of interest. If the absolute value of the corresponding correlation coefficient is below 0.5 the sensitivity is set to zero (we note that in such a case the obtained sensitivity is always low). This procedure is illustrated in Supplementary Figure 2 for the pools of 3PGA and DHAP. For instance, the relative change of 3PGA has largest effect (as quantified by the absolute value of the slope of the fitted line) on the relative change in the flux to starch, while the relative change of DHAP has very small effect on the relative change in this flux.

## Results

### Model scenarios

By using the described set-up (see Materials and Methods), here we compared three scenarios, referred to as Scenarios A, B, and C.

In Scenario A the compartmentalized contents were not treated as parameters to be optimized, i.e., they were fixed to the measured values. To this end, the error function to be optimized is given by *VWSS_t_* (see Materials and Methods). Therefore, for Scenario A, the model included 24 parameters, of which 11 described the steady-state flux distribution and remaining 13, the inactive fractions, leading to 73° of freedom (as there are 98 data points used) (Supplementary Table 3).

In contrast, in Scenario B, the compartmentalized contents were included as parameters to be optimized in the error function *VWSS_all_* (see Materials and Methods). Hence, additional 18 pool sizes appeared as parameters for which corresponding data points were used (see Supplementary Table 3), resulting in 73° of freedom. The findings from Scenarios B were the basis for conducting a sensitivity analysis which facilitates the investigation of the effect of pool size measurements.

In addition, we considered a third scenario, referred to as Scenario C, in which the estimated pool sizes are not required to fall within a pre-specified interval obtained from the measured data (but were bounded from below by 0.00001 and from above by 40000 C-atoms per gFW). In this case only ratios of fluxes and pool sizes can be estimated (see introduction). Therefore, the pool sizes of the pools of 3PGA, DHAP, and 2PGA are fixed to the measured values; in addition, the exchange fluxes between these pools are fixed to the maximum value used during the optimization. As a result, the pools act as one combined pool with a fixed size. This procedure allows the normalization of the fluxes and pool sizes during the optimization, but considers the presence of the mentioned combined pool. The assumption is driven by the observation that 3PGA, DHAP, and 2PGA show similar time-course of the unlabeled fraction. This observation can be explained either by the assumption of a combined pool or by negligible small pool sizes of DHAP and 2PGA. Moreover, in Scenarios A and B they are estimated to be close-to-rapid equilibrium. Like in Scenario A, the error function to be optimized is given by *VWSS_t_* (see Materials and Methods). Careful testing indicated that, in Scenario C, it is not possible to find a unique optimal flux distribution for the flux modes concerning the synthesis of starch, sucrose and trehalose; however, the ratio of the flux of photorespiratory pathway to the gross fixation could be reliably quantified.

In doing the statistical analysis, we were aware that the assumptions used in the modeling as well as any systematic errors in the evaluation of the time-course data (e.g., too slow quenching) or the metabolic content (e.g., due to metabolic losses) directly affected the goodness-of-fit. Although the treatment of the pool sizes as optimization parameters in Scenarios B resulted in a smaller error in comparison to that in Scenario A (109.6 and 126.4, respectively), the value did not fall in the statistically acceptable interval (95% confidence) of 54–94 (with 73° of freedom). However, the value of the error for Scenario C, in which the pool size parameters were practically unbounded (see Supplementary Table 3) was 2-fold lower, 62.3, and fall in a statistically acceptable interval of 42–78 (with 59° of freedom).

The effect of the three scenarios on the time-course error, *VWSS_t_*, per metabolite is shown in Supplementary Table 4. In Scenario C, the drop in *VWSS_t_* for ADPG is due to neglecting the measurement in the third time point of ADPG (marked in Supplementary Figure 3) in the fit (inclusion of this point did not have a strong effect on the ratio of photorespiratory flux to gross fixation, although it slightly increased the overall *VWSS_t_* error). Across all scenarios, FBP and Tre6P has the largest contribution to the overall *VWSS_t_*. This is due to the slower decay of the unlabeled fractional content of FBP in comparison to the metabolites in the CBC, which we speculate to be caused by the involvement of FBP in metabolic channeling (Winkel, 2004).

### Flux estimates for the three scenarios

As indicated in the values reported in Table 1, fluxes of starch and sucrose synthesis were slightly increased by 10–14%, while photorespiratory flux (i.e., rate of releasing carbon atoms via photorespiration) was slightly decreased by 3% in Scenario B in comparison to Scenario A. The flux of the synthesis of trehalose remained unchanged.

**Table 1:** **Flux estimates in Scenarios A and B**.

**Net flux**	**Value of net flux (nmol gFW^−1^sec^−1^C atoms)**					
	**Scenario A**			**Scenario B**		
	**Opt**	**Lower**	**Upper**	**Opt**	**Lower**	**Upper**
Starch synthesis	2.39	1.23	3.91	2.73	1.45	5.1
Sucrose synthesis	6.99	4.93	9.15	7.71	4.71	9.68
Photorespiration	3.93	3.07	5.06	3.81	2.94	4.91
Trehalose synthesis	0.00059	0.00031	0.00092	0.00059	0.00027	0.00092
Gross C fixation	13.31	10.94	16	14.25	11.66	16.65
Net C fixation	9.37	7.3	11.59	10.44	8.3	12.32
**Exchange flux**	**Value of exchange flux (nmol gFW^−1^sec^−1^C atoms)**					
3PGA ↔ DHAP	Inf.	Inf.	Inf.	Inf.	Inf.	Inf.
G6P*pl* ↔ G1P*pl*	Inf.	0	Inf.	Inf.	0	Inf.
F6P*cyt* ↔ G6P*cyt*	4.91	0.72	10.51	5.77	1.86	10.17
G6P*cyt* ↔ G1P*cyt*	Inf.	21.12	Inf.	244.99	0	Inf.
G1P*cyt* ↔ UDPG	Inf.	0	Inf.	8.76	0	Inf.
Ser ↔ Glyc	Inf.	0	Inf.	Inf.	0	Inf.
3PGA ↔ 2PGA	31.95	9.38	Inf.	64.7	14.49	Inf.
Error	126.43	–	–	109.59	–	–

*Estimates of fluxes via each mode and exchange fluxes. (A) The pool sizes were excluded from the optimization. (B) The pool sizes were included as parameters in the optimization and were required to fall in the measure interval (see Supplementary Table 3). The flux of photorespiration is denoted by released carbon atoms. The optimal fit is indicated by opt, and the error of the fit indicates the variance-weighted sum of squares VWSS_t_ for Scenarios A and VWSS_all_ for Scenario B (see Materials and Methods). The lower and upper confidence limits (95%) were obtained by Monte-Carlo simulation, indicated in the column titled lower and upper. The upper bound of an exchange flux used during the optimization was 245, indicated by inf. The plastidic and cytosolic pools of a compartmentalized metabolite are indicated by pl and cyt. Most exchange fluxes are not identifiable and/or are high*.

In Scenario C, the ratios of the fluxes the net C fixation, the synthesis of starch, sucrose, and trehalose, could not be uniquely determined within a meaningful range. We argue that the issue of unidentifiability is largely due to the nature of the branched chain with two branching points. The first branching point appears at the splitting between the synthesis of starch in the chloroplast and the flux of the synthesis of sucrose and starch in the cytosol, while the second is at the splitting between the synthesis of sucrose and trehalose. In contrast, the ratio of photorespiration is resolved at the merging point as the best-fitting flux ratio between the influxes from the pools of RuBP and Glyc to the pool of 3PGA. The observed ranges of the flux estimates are shown in Table 2 as fractions of the gross fixation. Table 2 also includes an optimal fit and the observed ranges obtained from a set of fits, which have an error lower than an indicated boundary. We would like to note that the chosen boundaries for the error fall in the statistically acceptable interval of 43–79. The relative amount of photorespiration to gross C fixation could be estimated to be between 16 and 22%. While the fluxes of the synthesis of sucrose and trehalose range from 0 to 82% and 83%, respectively, of gross fixation, the flux toward starch reaches from 0 to 50% and the sum of the fluxes toward sucrose and trehalose reaches from 30 to 83%. Moreover, we further investigated this effect by fixing the modes of the synthesis of trehalose and sucrose to a very low value (0.0001) and fitting the model to the data. In this case, the lowest observed error was around 80, i.e., 130% higher than the lowers observed error in the original setting. Therefore, we concluded that an optimal fit of our model to the experimental data requires a minimum flux in the cytosolic branches.

**Table 2:** **Estimates via each net flux mode as fraction of gross carbon fixation in Scenario C**.

**Net flux mode**	**Fraction of gross C fixation (Scenario C)**				
	**Max error 63**			**Max error 65**	
	**Opt**	**Lowest**	**Largest**	**Lowest**	**Largest**
Starch synthesis	0.28	0.00	0.51	0.00	0.70
Sucrose synthesis	0.23	0.00	0.82	0.00	0.82
Photorespiration	0.20	0.16	0.22	0.15	0.23
Trehalose synthesis	0.29	0.00	0.83	0.00	0.83
Cytosolic flux	0.53	0.30	0.83	0.12	0.84
Gross C fixation	1.00	1.00	1.00	1.00	1.00
Net C fixation	0.80	0.78	0.84	0.77	0.85

*The values in the column indicated by opt correspond to the minimal error of 62.3. The additional column show the ranges of the obtained flux estimates with a corresponding error lower than 63 and 65, respectively. The term “Cytosolic flux” refers to the sum of the fluxes of the synthesis of sucrose and trehalose*.

We speculate that this finding is due to the use of the pool sizes as weights in the observation model which affect the flux ratio at this branching point between cytosol and chloroplast. The decays of the unlabeled fractional content of FBP and F6P are rather slow in comparison to the decay of the CBC intermediates (Szecowka et al., 2013), since the fluxes in the CBC are larger and the CBC reactions are included in all flux modes (see Supplementary Figure 1). The assumption that the observed slow decay is caused by the cytosolic pool, would imply that this pool is to be large enough to constrain the ratio between the cytosolic and plastidic pool sizes. In addition, a larger flux toward starch in the plastid would demand a larger pool size of the plastidic pool to explain the data and, due to the constrained ratio of the pool sizes, a larger pool in the cytosol. On the other hand, a larger flux ratio toward starch is accompanied by a smaller flux in the cytosol toward sucrose and trehalose, which demands a smaller size of the cytosolic pool to explain the data. This contradiction imposes a lower limit to the flux through the cytosol. However, we assume the effect to be low, since, as indicated in Table 2, the set of fits with a maximal error of 65 shows a minimum cytosolic flux of 12% of the net fixation.

In all scenarios some pools are estimated to be in rapid equilibrium, indicated by the high exchange flux between the pools in the optimal fit (see Table 1 and Supplementary Table 5). Such pools can be considered to affect the dynamics of the labeling as one combined pool. We referred to such a combined pool by its components as, e.g., 3PGA-DHAP-2PGA. Several other pools are indicated be close to rapid equilibrium, namely, Ser-Glyc, G6P-G1P in the chloroplast and G6P-G1P-UDPG in the cytosol. We would like to indicate that the latter pools do not necessarily have to be in rapid equilibrium, since the exchange flux are mostly unidentifiable. Nevertheless, the issue of unidentifiabliliy corresponds to the absence of a delay between the time-course of these pools. Therefore, a combined pool have to be understood as a set of connected pools showing a similar labeling time-course, either due to a large exchange flux or to small sizes of the downstream pools.

Due to the findings described above, only the ratio between the flux of photorespiration (carbon atoms released) and gross carbon fixation can be compared in the different scenarios. In Scenario C this ratio could be estimated to ~20% with a 95% confidence interval of 5–34%. The corresponding optimal value in Scenario A was 30% with a confidence interval of 24–36%, while in Scenario B, the value was 27% with a confidence interval of 21–33% (see Table 3). Alternative estimates of the rate of photorespiration cover a range from 13 to 33% of the gross fixation in different species and depend on the applied method (Sharkey, 1988; Busch, 2013). Kinetic modeling of the carboxylation and oxygenation of RuBP conclude to a carbon release of ~26% of the net fixation (21% of gross fixation) and consider the partial pressure of CO_2_ in the leaves to be about 60–70% of the one in air (Sharkey, 1988). Since we have no direct access to the partial pressure in our system we rely on the general assumption of such a partial pressure in C_3_-plants. Investigation of the time-course of the ^13^C enrichment of CBC intermediates in soy bean estimate the rate of photorespiration to be 21% of the net C fixation (17% of gross fixation) (Cegelski and Schaefer, 2006). The study aimed at quantifying the ratio between newly fixed carbon atoms and the backflow of unlabeled atoms from photorespiration intermediates to the intermediates of the CBC. In summary, the consideration of the pool sizes in Scenario C as unbounded parameters to be optimized resulted in flux ratio estimates which were in better agreement with benchmark value for the ratio of photorespiration to gross fixation. We argue that in this case the ratio is resolved at the merging point as the best-fitting flux ratio between the influxes from the pools of RuBP and Glyc to the combined pool of 3PGA-DHAP-2PGA, while in the Scenarios A and B the fluxes are more strongly determined by the measurements of the pool sizes of the intermediates of the photorespiratory pathway (i.e., Gly, Ser, Glyc).

**Table 3:** **Estimates via each net flux mode as fraction of gross carbon fixation in Scenarios A and B**.

**Net flux mode**	**Fraction of gross C fixation**					
	**Scenario A**			**Scenario B**		
	**Opt**	**Lower**	**Upper**	**Opt**	**Lowest**	**Largest**
Starch synthesis	0.18	0.09	0.27	0.19	0.11	0.35
Sucrose synthesis	0.53	0.42	0.62	0.54	0.36	0.63
Photorespiration	0.30	0.24	0.36	0.27	0.21	0.33
Trehalose synthesis	0.000044	0.000021	0.000070	0.000041	0.000020	0.000069

*The flux of photorespiration refers to released carbon atoms. The optimal fit is indicated by opt, and the error of the fit indicates the variance-weighted sum of squares VWSS_t_ for Scenario A and VWSS_all_ for Scenario B (see Materials and Methods). The lower and upper confidence limits (95%) were obtained by Monte-Carlo simulation, indicated in the column titled lower and upper. The plastidic and cytosolic pools of a compartmentalized metabolite are indicated by pl and cyt*.

We note that the estimates of net carbon fixation in Scenarios A and B are 2-fold smaller in comparison to those reported from experiments under similar conditions (Florian et al., 2014; Sulpice et al., 2014). The reasons for the underestimation could be due to the lower irradiance in the labeling box, not considering the time needed to replace ^12^CO_2_ in the leaves with ^13^CO_2_, and to the combined effect from possible underestimation of pool sizes for some metabolites (see Section Pool Size Estimates, below). An underestimation of the net fixation can also explain the underestimation of the ratio of photorespiration.

### Pool size estimates

The optimal values of the compartmentalized content of Scenario B together with the confidence intervals from the Monte Carlo simulation (see Methods) were provided in Table 4 (illustrative summary of the Monte Carlo simulation results could be found in Supplementary Figure 4). The compartmentalized content was divided into inactive and active pool, shown in Supplementary Tables 6, 7, respectively. The optimal values for the inactive fraction in the three considered scenarios were almost equal and the corresponding confidence intervals were largely overlapping for Scenarios B and C. Notable differences in Scenario C were observed for 3PGA, DHAP, and 2PGA, for which larger inactive fractions were estimated. Therefore, the major differences in the estimates of the fluxes and the reduction in the total error, in Scenario B, and the error due to fitting the time courses, in Scenario C, could be attributed to the estimation of the compartmentalized content or to the size of the active pool, which are, for the purpose of comparison of different scenarios, proportional. Therefore in the rest of this study we will use the term (active) pool size to mean compartmentalized content.

**Table 4:** **Estimates of metabolic content**.

**Pool**	**Compartmentalized content *c* (nmol gFW^−1^ C atoms)**				
	**Measured**		**Optimized (Scenario B)**		
	**Mean**	**Std**	**Opt**	**Lower**	**Upper**
3PGA	600.3	135	873.6	687	1038.9
DHAP	47.6	1.8	47.7	44	51.5
FBP*pl*	37.5	9.7	36.8	19.4	56.7
F6P*pl*	176.2	29.8	171.6	111.4	227.3
G6P*pl*	176.4	52	173.2	89.5	277
G1P*pl*	5.6	1.1	5.7	3.9	7.8
ADPG	3.3	0.3	3.3	2.7	3.9
FBP*cyt*	16.1	4.1	14.4	4.5	23.1
F6P*cyt*	342	57.8	265.5	114.6	372.4
G6P*cyt*	861.1	254	995.2	459.8	1353.7
G1P*cyt*	64.5	13.3	63	31.4	87.7
UDPG	214.5	34.2	218.2	150.8	278.1
Suc6P	9.8	4.3	7.9	2.5	17.9
Tre6P	1.9	0.5	1.9	0.9	3
Gly	1086.3	118	1102.3	843.7	1343.6
Ser	12793.9	978	12047.2	10014.7	13799.1
Glyc	506.5	195	477.8	149.4	843.4
2PGA	60	13.5	63.2	39.3	90

*The table contains the measured values of the compartmentalized metabolic content *c* and the respective optimized values in scenarios B. The compartmentalized metabolites in the cytosol and chloroplast, indicated by the subscript c and p, were determined from the measured total metabolic content by splitting them according to the ratio given by the n.a.f. data. The corresponding standard deviations were calculated such that their ratio equals the ratio of the pool sizes and the sum of the variances equal the total measured variance. Variances for the n.a.f. data are not reported by the existing computational tools. This compartmentalized metabolic content is the sum of the active and inactive pool. The optimal fits are denoted by opt. The lower and upper confidence limits (95%) were obtained by Monte–Carlo simulation. All values are denoted in nmol gFW^−1^ C atoms*.

**Figure 1:**
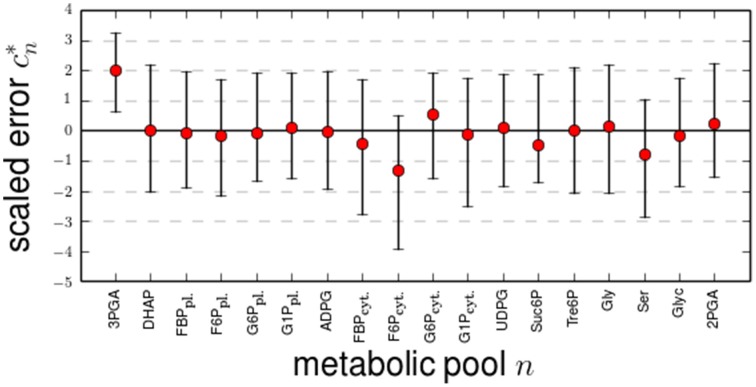
**Changes of optimized metabolic content in Scenario B**. The differences (errors) between the optimized metabolic content *c_sim, n_* (red circle) as well as their lower and upper 95%-confidence limits (error bars) to the measured values *c_obs, n_* were scaled by the measured standard deviation by $c_{obs.,\;n}^{*}\;=\;\frac{c_{obs,n}-c_{sim,\;n}}{\sigma_{c_{obs,\;n}}}$. All changes of the values by the optimization can be explained by measurements errors. Note that for the assumed normal distribution of the measurement errors, the 95% confidence intervals corresponds to 2 σ*_c_obs., n__*.

We visualized the estimates of the compartmentalized content of Scenario B relative to the measured values weighted by the respective standard deviations. This visualization helps in illustrating the likelihood that the optimized value can be explained by the assumed normal distribution of the measured values. In Scenario B the optimized values were well within the range of the normal distribution with means corresponding to the measured values of the pool sizes with three notable exceptions: As shown on Figure 1, the pools of 3PGA and Ser as well as the pool of F6P in the cytosol exhibited the largest differences in comparison to the measured values. The remaining estimated pools only slightly changed away from the experimentally determined values. A change in a pool size in comparison to the measured values during optimization has two effects on the variance-weighted sum of squared error in Scenario B: First, this change always directly contributes to the error of the metabolic content *VWSS_c_*; second, it can influence the time-course of the unlabeled content by introducing a delay if the pool size is not too low. If a pool has no influence on the time-course of the unlabeled content, it has no influence on the time-course error, *VWSS_t_*, and thus it only contributes to the error of the content. In this case, the optimized value must be equal to the measured value. Conversely, each optimized pool size which differs from the measured value must affect the time-related error.

**Figure 2:**
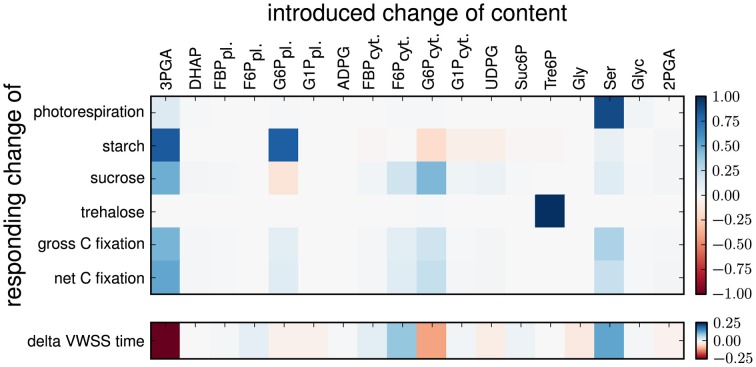
**Sensitivities of flux estimates and time error to perturbation in metabolic content for scenario B**. The heatmap shows the relative changes in flux estimates and time error upon relative changes in metabolic content in Scenarios B. The content of a selected metabolic pool was changed and fixed and the model was re-optimized for the remaining parameters (see Material and Methods).

To further determine the effect of the changes to the optimized values for the metabolic content in scenarios B, we conducted a sensitivity analysis for the time-related error and the flux estimates by perturbing the optimal pool sizes. As shown in Figure 2, the flux estimates were most sensitive to changes in the optimal value of the pool sizes for 3PGA, G6P in cytosol and plastid, F6P in cytosol, Tre6P, and Ser. In addition, as shown in Supplementary Figure 5, illustrating the delay between the time course of the unlabeled content and the time course of newly synthesized unlabeled molecules for the investigated Scenario B, these six pools introduced a large delay. Moreover, a delay is not observed between the members of the combined pools: 3PGA-DHAP-2PGA, Ser-Glyc, G6P-G1P in the plastid and G6P-G1P-UDPG in cytosol. We conclude, that in Scenario B the absolute fluxes are mainly resolved by the pool size, which are large enough to introduce a delay to the time-courses. Remarkably, the pool sizes of Suc6P and ADPG are too small to introduce a delay and therefore do not contribute to the determination of the fluxes in the corresponding pathways. The effective size of a combined pool in rapid equilibrium is then given by the sum of the involved pool sizes. The corresponding error of the effective pool size (change of pool size by optimization) is given by the sum of the errors of the involved pool sizes, while the ratio of the single errors is determined by the ratio of the corresponding variances to the sum of all variances. If these variances differ strongly, the size of a combined pool is altered mainly due to the change of the pool with the largest variance. This explains why, for instance, the size of the combined pool 3PGA-DHAP-2PGA is change by the size of the pool of 3PGA, while the size of the pools of DHAP and 3PGA remain unchanged.

Since in Scenario C the fluxes of the net C fixation cannot be determined, the compartmentalized content and the active pool sizes also cannot be estimated. The observed ranges of the compartmentalized contents range from very small values (often the lower boundary used during the optimization) to the maximum (see Supplementary Table 5). This is caused by two effects. First, the set of optimal fits comprise estimates with different distributions of the three fluxes of the net fixation, which are accompanied by different estimated (or ranges of estimates) of the pool sizes. Since the time constants are determined by the data, a higher flux is accompanied by a higher pool size. While the lowest observed sizes of pools in the chloroplast are identical with the lower boundaries used in the optimization, the lowest sizes of the pools in the cytosol are typically higher. This is in agreement with the finding of a minimum flux though the cytosol and most evident for the pool of UDPG. Second, even for a given distribution of the net fluxes, there can be different distribution of exchange fluxes due to the reversibility of the reactions and the associated exchange fluxes, which corresponds to different best-fitting pool sizes. The relation between exchange flux and pool size can be demonstrated by the estimates of the photorespiratory pathway, since the flux through this pathway is determined in Scenario C. The pool size of Gly, as an intermediate of the pathway, is estimated within certain boundaries (see Supplementary Table 5), while the pool size of Ser and Glyc range from zero to and the upper boundary. The time-course of Ser and Glyc are quite similar, and both show a delay to the time-course of their precursor Gly. Two modeling assumptions can explain this observation: (1) The delay to Gly is explained by the pool size of Ser, while the pool size of its downstream Glyc is too small to introduce a further delay. In this case, the exchange between the latter two can have any value and the reaction might be irreversible (Supplementary Figure 5C); (2) The pool size of Ser can be very small, but Ser is in rapid equilibrium with a large pool of Glyc such that they influence the time-course as one combined pool. The size of the combined pool is then equal to the size of the pool of Ser in the first case. Therefore, the sum of both pools can still be determined from the data (see Supplementary Table 5, Supplementary Figure 6). We note that, in principle, in cases with high exchange fluxes, the pool sizes or their ratios could be estimated from the time-courses, but this demands the resolution of the different involved time-constants from the time-course data; the latter is strongly limited by the number of time points and the precision of the measurements. We note that similar considerations hold for the combined pools in cytosol and chloroplast, but are here not in the scope of interest.

The unidentifiablity of the distribution of the net fluxes and the corresponding pool sizes in Scenario C handicaps the comparison of the scenarios. Nevertheless, in Scenario C the ratio between the the pool size of Gly and the sum of Ser and Glyc was obtained. While this ratio is ~0.025 in Scenario A and 0.027 in Scenario B, the value of 0.56 in Scenario C allows an optimal fit to the time-course data (see Supplementary Table 4, Gly). However, it is questionable whether this due to measurements error of the content or to incompleteness of the pathway model.

### Sensitivity analysis

Sensitivity analysis revealed that the metabolic content of 3PGA had the strongest effect on the time-related error in Scenario B (see Figure 2). Perturbation of the optimal value of 3PGA mainly affected the flux of the synthesis of sucrose and starch. Our findings indicated that the increased pool of 3PGA increased the delay in both 3PGA and DHAP, and therefore, resulted in a better fit to the data, as shown in Supplementary Table 4 supporting the strong reduction in time-course error for 3PGA and DHAP in Scenario B. Moreover, this enables a higher flux through these pools and finally leads to an increase in the estimated net fixation. While the effect can still be explained as a measurement error of the metabolic content, it could also point to the possibility of an increased effective pool size. For example, intermediates of the CBC or glycolysis could contribute to the effective pool size, due to reversibility of the reactions of both pathways (Raines, 2003; Plaxton and Podestá, 2006).

The pool size of Ser, expectedly, exhibited the largest effect on the photorespiratory flux in Scenarios B (see Figure 2). The decreased pool size of Ser in Scenarios B was accompanied by decrease in the flux via photorespiration. This provided a better fit to the data of Ser and Gly, although the simulation indicated that the decay was still too large (Supplementary Figures 5), reflected in the relatively small effect on the time-course error of these metabolites (Supplementary Table 4). Remarkably, in Scenario C the time-course error of Gly is strongly reduced in comparison to the other scenarios and the ratio of Gly and Ser (Ser-Glyc) is strongly changed toward Gly. A larger pool of Gly can introduce the observed delay in the time-course of this pool. Alternatively, the delay can be affected by the pools of glycolate and glyoxylate, which were not considered in the pathway model, or by discrepancy from the random labeling assumption. However, in such a case the size of the pool of Ser would additionally need to be reduced to fit to the time-course data of Ser.

The sensitivity analysis indicated that perturbation of the optimized values of the plastidic G6P pool had the strongest effect on the flux of starch synthesis, while changes to the cytosolic G6P and F6P pools largely affect the flux toward sucrose. These pools introduce a strong delay to the time-course (Supplementary Figure 5).

We speculate that the time-courses of the unlabeled content of G6P and G1P was mainly dominated by the cytosolic part, since these pools were, according to n.a.f. data, larger than their counterpart in the plastid. The assumption is further supported by the observation that the unlabeled content of ADPG decays faster than the unlabeled content of G6P and G1P. The unlabeled metabolic fractions of G6P and G1P in the plastid cannot decay faster than the one of their downstream product ADPG. Therefore, the estimated flux toward starch synthesis was mainly determined by the pool size of G6P in the plastid and the time-course of ADPG, which was the only observed intermediate of this branch of the plastidic pathway. The optimization resulted in an estimate of the starch synthesis flux to better fit the time-course of ADPG and the measured pool sizes of the other intermediates of this branch, mainly G6P. In contrast, the flux toward the synthesis of sucrose was much more strongly determined by the time-courses of the hexose-phosphates. The decrease in the pool size of F6P and the increase in the pool size of G6P therefore provided a better fit to the observed delay in the time-courses. Following this reasoning, the flux toward sucrose was more strongly constrained by the data set.

Our sensitivity analysis (see Figure 2) demonstrated that the flux of the synthesis of trehalose was nearly exclusively influenced by the pool of Tre6P. In addition, perturbations of this pool size have small effect on the time-related error and, consequently, no effect on other flux estimates. These findings indicated that the pool size of Tre6P largely determines the flux of the synthesis of trehalose. The claim about the effect of the Tre6P pool size can be explained by the high overlap of the flux modes of the synthesis of sucrose and trehalose. The data of the overlapping (shared) intermediates resolve the sum of both fluxes. While the high flux toward sucrose is determined by the data of these shared intermediates, the much lower flux toward trehalose is not determined in the same way. In fact, small perturbations of the flux of the synthesis of trehalose have nearly no effect on the time-course of the shared intermediates. Moreover, the observed delay of the time-course of Tre6P determines the ratio between the flux of the synthesis of trehalose and the pool size of Tre6P. Since this flux is not fully determined by the labeling data of the shared intermediates, it largely depends on the pool size measurement of Tre6P.

## Conclusion

As demonstrated theoretically in the introduction, in the absence of alternative flux measurements, data on pool sizes are mandatory for the estimation of fluxes at divergent branch points from non-stat.-^13^C-labeling data. Pool size ratios can be provided to enable the estimation of flux ratios at branching points. We found, that pools, which introduce a delay in the time-course of the labeling process, are, therefore, of critical interest. In addition, factoring in information about the inactive pool sizes and their distribution across compartments will have an effect on the estimated fluxes. Here, for simplicity, we treated the total inactive pool equally distributed in the two compartments, although one could consider the more involved scenario of having two free inactive pools treated as parameters summing to a given number. Therefore, n.a.f. data about the inactive pools and more robust procedures for their measurement is expected to increase the reliability of the flux estimates.

The three considered scenarios were instrumental in determining the discrepancy between measured and estimated pool sizes. The findings from this analysis could be readily used to determine if there were systemic inaccuracies in the measurement of, usually large, pool sizes which would have to be addressed by development of new or improved measurement methods. On the other hand, since absolute fluxes could readily be determined by considering data about the size of the involved metabolic pools, the used *in silico* set-up shed light on the discrepancy between flux estimates in the three scenarios as influenced by the introduction of parameters to be estimated. More specifically, while the optimization of the pool sizes had a variable influence on the absolute values of the fluxes, depending on the constraints on the pool size estimates, the ratio of photorespiration to the gross fixation was closer to its existing estimates from other techniques under similar conditions (Sharkey, 1988; Cegelski and Schaefer, 2006).

In addition, the optimized pool sizes were the basis for a sensitivity analysis providing insights into sizes of particular metabolic pools which have strong effect on the flux estimates of canonical pathways. Although the analysis depended on a simplified model of central carbon metabolism, it was interesting to observe that the small pool of Tre6P was a key determinant of the flux to trehalose synthesis, while the sucrose synthesis rate is resolved by the delay of hexose-phosphates.

Altogether, our study points that pool size measurements of greater precision are needed to determine accurate flux estimates, especially for pathways emerging from a divergent branch point, irrespective of the method used. These requirements will become particularly relevant for flux estimation in secondary metabolic pathways which have such structure.

### Conflict of interest statement

The authors declare that the research was conducted in the absence of any commercial or financial relationships that could be construed as a potential conflict of interest.
