# Supplementary material for: Pool size measurements facilitate the determination of fluxes at branching points in non-stationary metabolic flux analysis: the case of Arabidopsis thaliana
Source: Front Plant Sci. 2015 Jun 2;6:386. doi: 10.3389/fpls.2015.00386 (PMC4451360; doi:10.3389/fpls.2015.00386)
Supplement: Supplementary file 1 [file Presentation1.PDF]

Frontiers in Plant Sciences

Research Article
20 May 2015

1

Supplementary Material: Effects of pool sizes on flux estimates in the central carbon metabolism of Arabidopsis

Robert Heise [1], Alisdair R. Fernie [1], Mark Stitt [1] and Zoran Nikoloski [1,*]

[1] *Max-Planck-Institute for Molecular Plant Physiology, Potsdam/Golm, Germany*

Correspondence*:
Zoran Nikoloski
Max-Planck-Institute for Molecular Plant Physiology, Am Mühlenberg 1, 14476
Potsdam/Golm, Germany, nikoloski@mpimp-golm.mpg.de

Large-scale metabolic network models.

1 SUPPLEMENTARY FIGURES

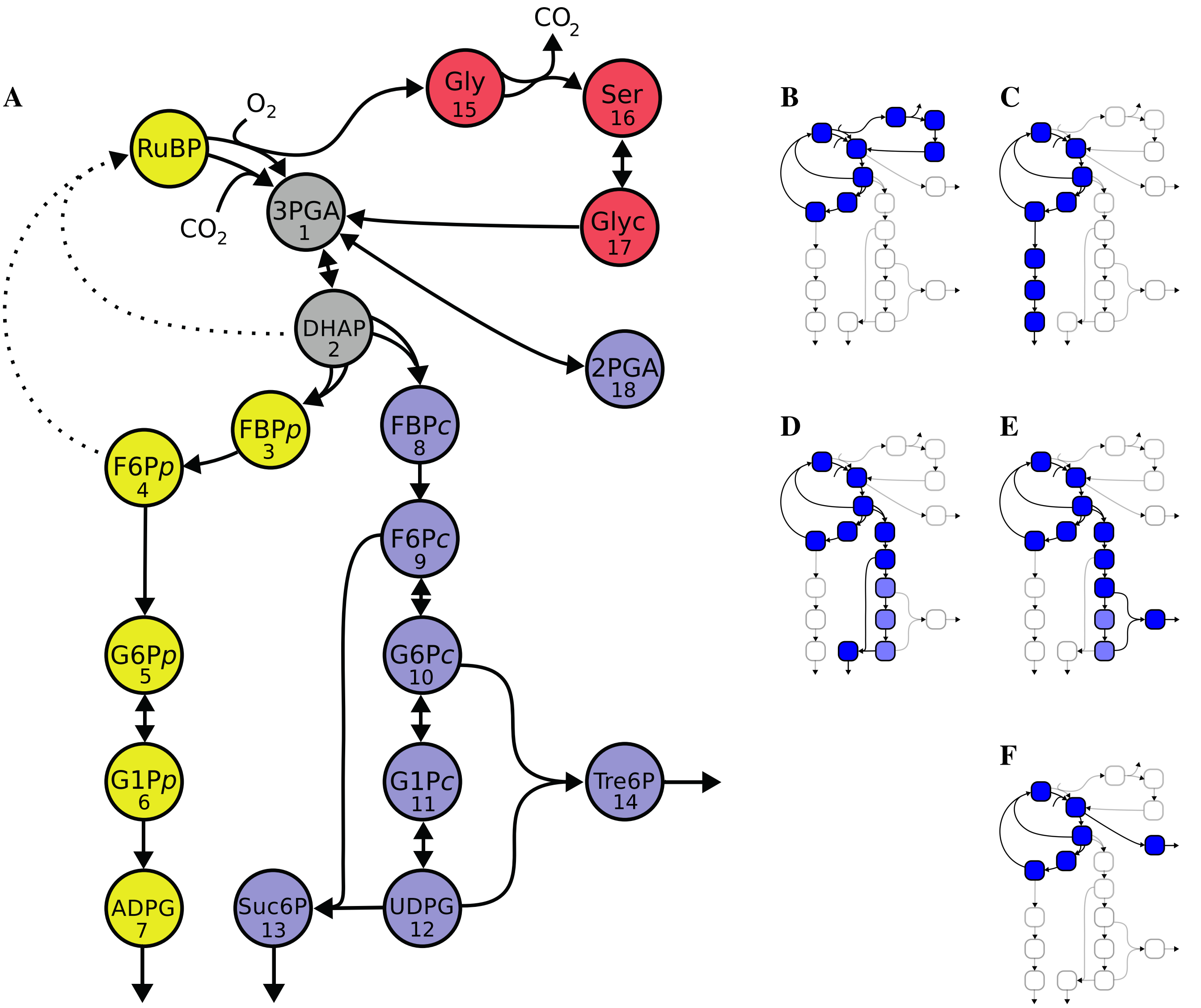

Supplementary Figure 1. Schematic representation of the pathway model and its modes. (A) Pathway model with metabolites of different compartments (yellow - chloroplast, blue - cytosol, red - intermediates of photorespiration represented by one pool for each, gray - 3PGA and DHAP of chloroplast and cytosol in rapid equilibrium). The italic letters *p* and *c* indicate the localization of the pool in plastid or cytosol. The numbering correspond to the indices of the variables in the description of the ODS system (see **Supplementry Equations**). (B-F) The five modes that generate the steady-state net flux distributions, **(B)** photorespiration, **(C)** starch, **(D)** sucrose, **(E)** trehalose, **(F)** amino acids. Each reversible reaction is additionally described by an exchange flux. In the actual implementation of the model, mode (F) is set to zero, which is depicted by the lack of an outwards arrow in (A).

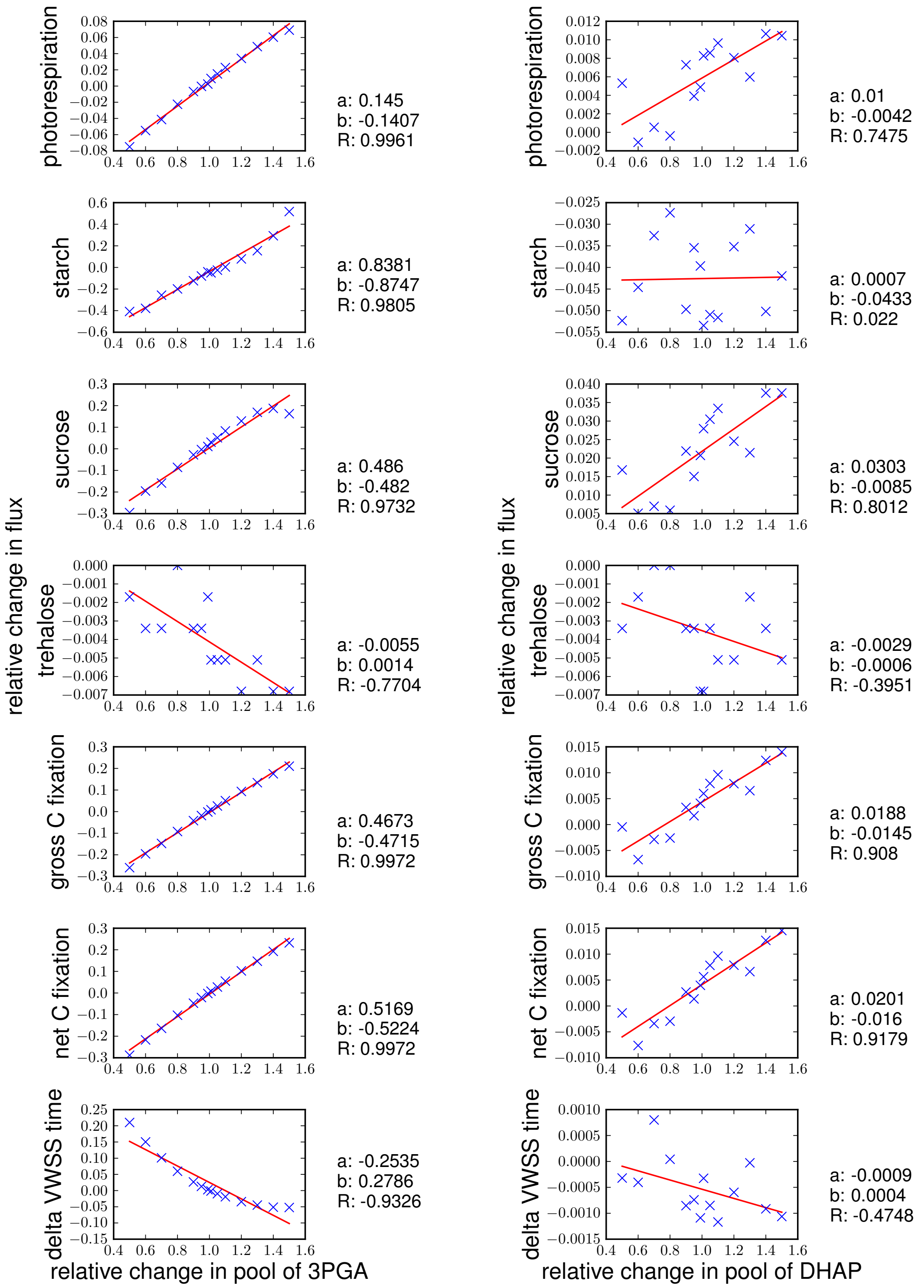

Supplementary Figure 2. Example illustration of the approximation of the sensitivities with respect to Scenario B. The figure shows relative changes of the flux estimates and the time-error upon fixed perturbation of the pool size (blue crosses). The perturbed pool size is fixed during the optimization. A linear regression (red line) provides the slope (a), the intersection (b) and correlation coefficient (R). The slopes approximate the sensitivities, if the obtain data shows a linear correlation between change in relative change in pool size and relative change in the estimates flux. In the non-correlated case, the slopes are small, probably due to numerical issues of the optimization and the corresponding stopping criteria. The left side shows the analysis for the pool 3PGA, which has a high influence on the flux estimates. The right side shows the analysis for DHAP, which has a low influence.

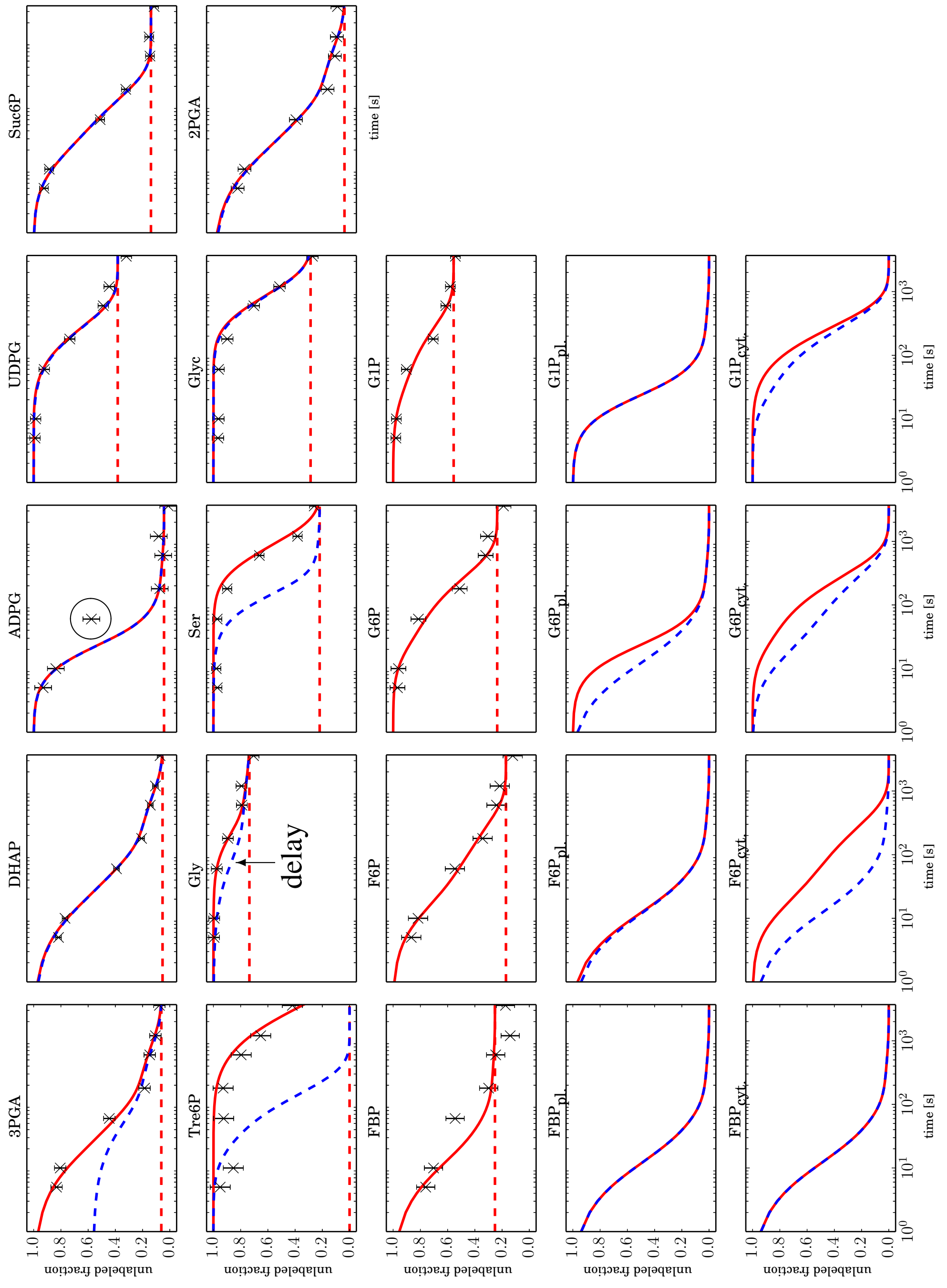

Supplementary Figure 3. Representive simulation of a set of best-fitting time-courses of the unlabeled fractions in Scenario C. The simulation of best fitting the time-course of the unlabelled content (red line) and the experimental obtained values (crosses) including the standard deviations (error bars) are shown for all measured metabolites. The amount of the inactive pool is indicated by the red dashed line. For the compartmentalized metabolites, the time-course of the fraction of newly synthesized unlabelled molecules (blue dashed line) is included to the pictures. For the compartmentalized hexosephosphates the time-courses of the unlabelled fraction of the active pool is additionally shown in order to illustrate the delay introduced by the corresponding pool. The circle marks the time-points of ADPG, which is not considered in Scenario C. The arrow indicates the delay introduced by the pool of Gly, which was not observed in Scenario A and B and is accompanied by a better fit of Gly to the time-course data.

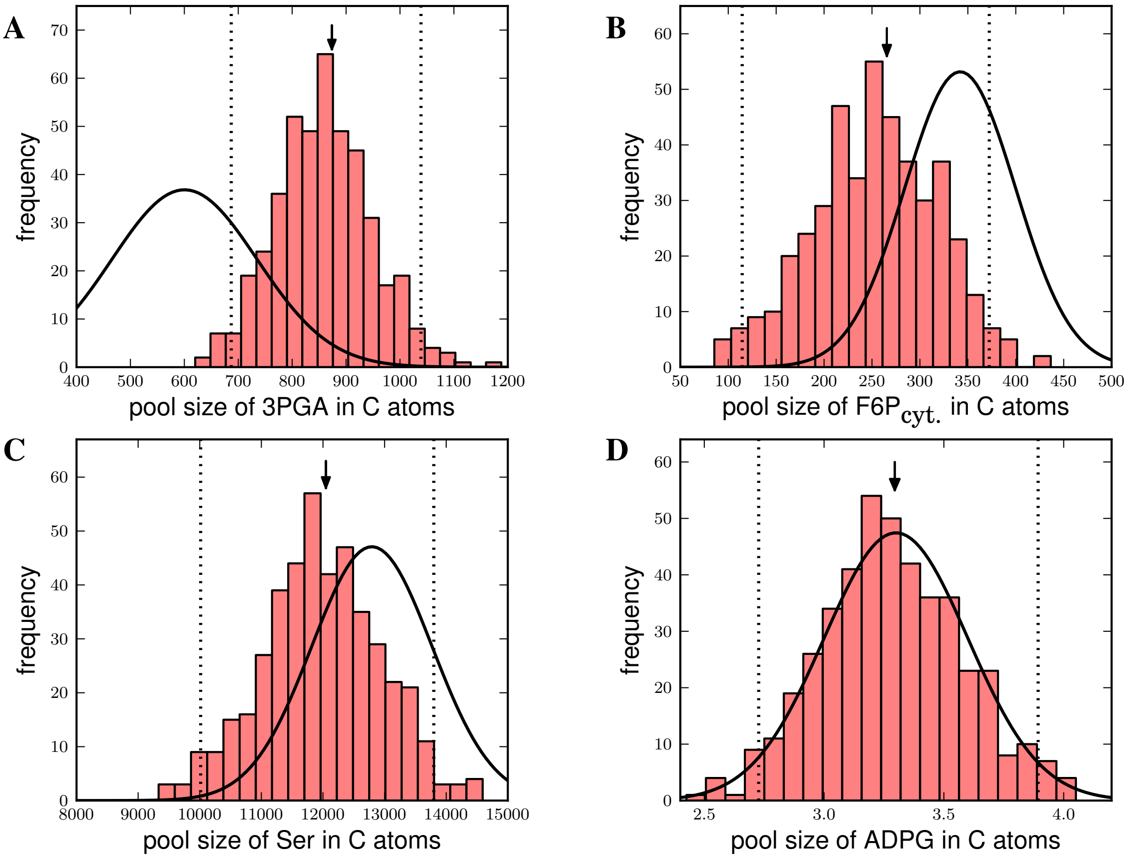

Supplementary Figure 4. Histograms of Monte-Carlo simulation. The figure compares the assumed normal distribution of the measurements of the ”compartmentalized” metabolic content (black line) and the histogram obtain by the Monte-Carlo simulations (red bars). The arrow indicates the optimal. The confidence intervals (95%) are indicated by the dashed lines. **(A)** 3PGA, **(B)** F6P in the cytosol, **(C)** Serine and **(D)** ADPG. The pool of ADPG is not change by the optimization. The histogram mainly resamples the measured distribution.

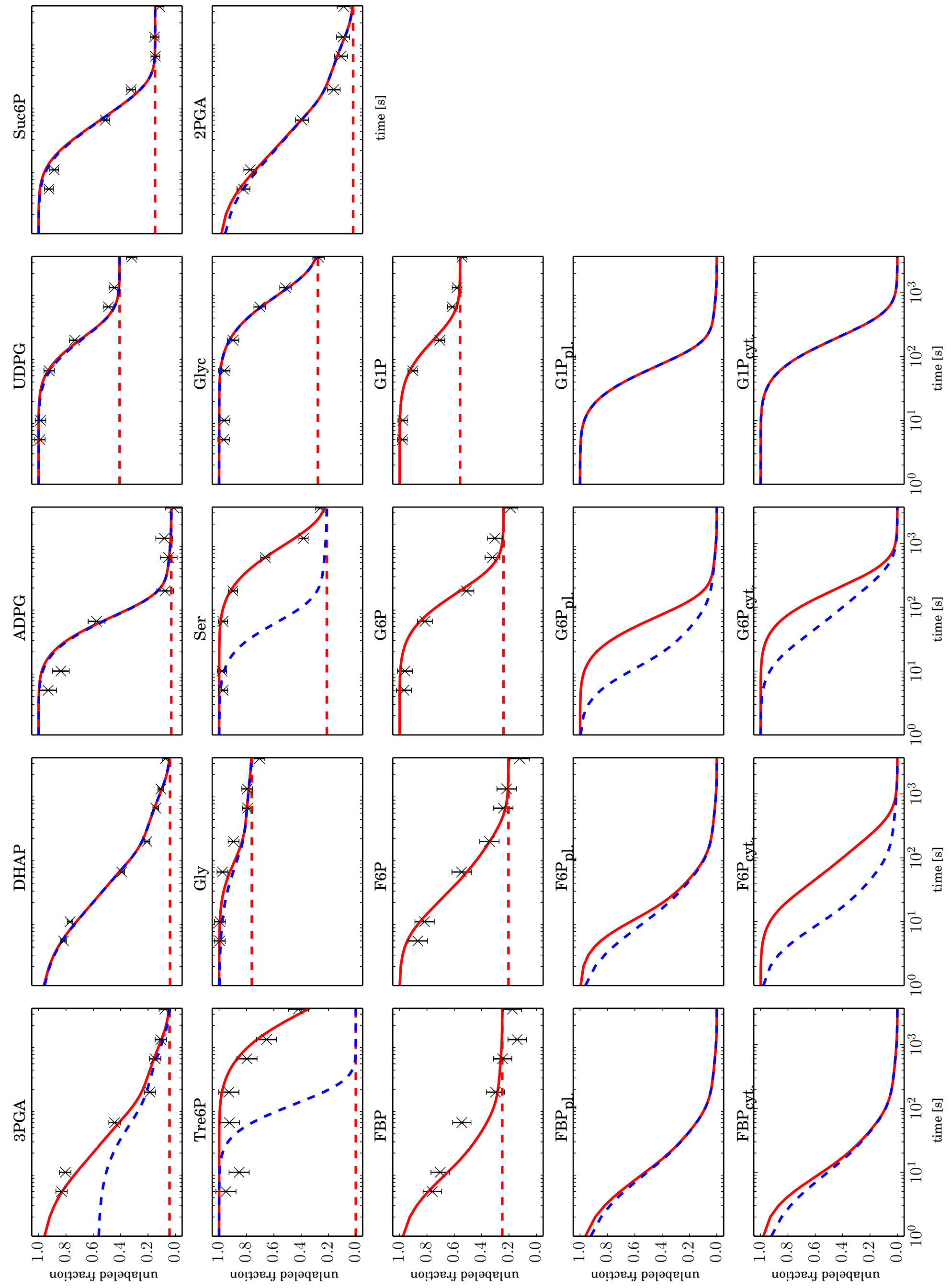

Supplementary Figure 5. Simulation of the best-fitting time-courses of the unlabeled fractions in Scenario B. The simulation of best fitting the time-course of the unlabelled content (red line) and the experimental obtained values (crosses) including the standard deviations (error bars) are shown for all measured metabolites. The amount of the inactive pool is indicated by the red dashed line. For the compartmentalized metabolites, the time-course of the fraction of newly synthesized unlabelled molecules (blue dashed line) is included to the pictures. For the compartmentalized hexosephosphates the time-courses of the unlabelled fraction of the active pool is shown in order to illustrate the delay introduced by the corresponding pool.

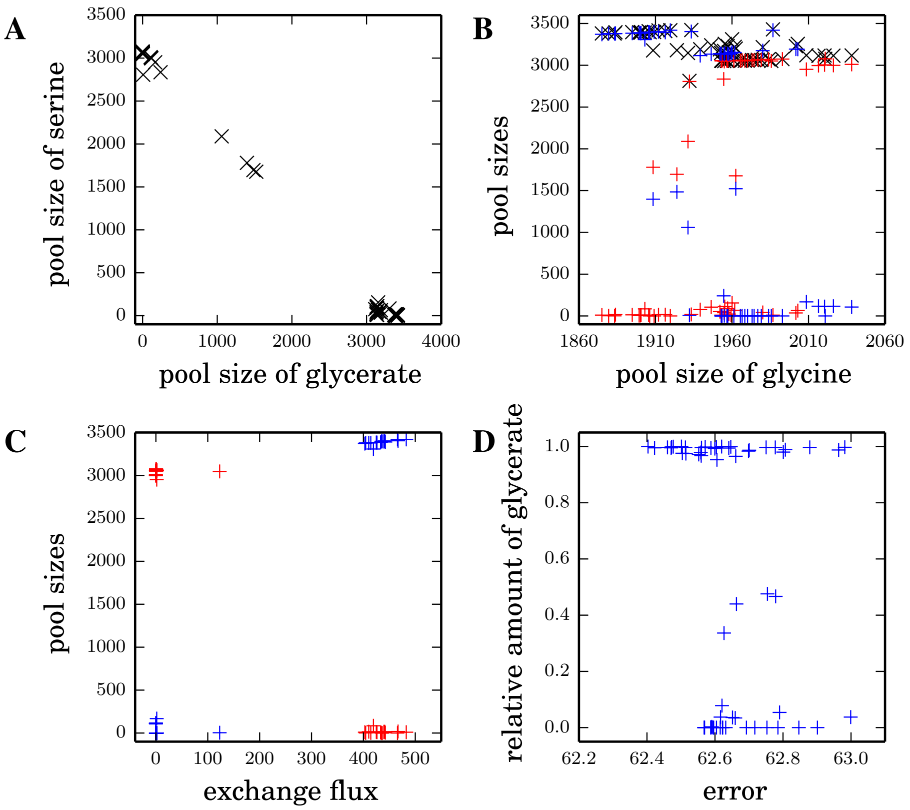

Supplementary Figure 6. Relation between best fitting pool sizes and exchange fluxes of intermediates of photorespiration in Scenario C. All data points shown refer to the set of best-fitting parameter with an error smaller than 63 and are normalized to the corresponding estimate of the photorespiratory flux in the set. **(A)** Two clusters of estimates of pool size of serine and glycerate: The most estimates of the pool sizes of serine and glycerate belong to one of two clusters, in which one of the pools has a size very close to zero and the other falls in the interval from 2700 to 3500. The pool sizes of the remaining estimates sum up to be in this interval. **(B)** Ratio between estimates of the pool size of glycine and the sum of the pools of serine and glycerate: The figure shows the two clusters of the pool sizes of serine (red crosses), the corresponding two clusters of the estimates for the pool size of glycerate (blue crosses) and the sum of both as black crosses. The figure illustrates the level of determination/variation of the shown pool sizes in comparison the photorespiratioy flux. **(C)** Relation between the best-fitting pool sizes of serine and glycerate and corresponding the exchange flux of the reaction: ser $\leftrightarrow$ glycerate. The figure shows two scenarios corresponding to the two clusters: 1) The pool size of serine (red crosses) is high and the pool size of glycerate (blue crosses) is close to zero. In the case the exchange flux is close to zero. This indicates an irreversible reaction ser $\rightarrow$ glycerate. 2) The pool size of serine is close to zero and the pool size of glycerate is high. This case is accompanied by a high exchange flux and indicate a rapid equilibrium between serine and glycine. **(D)** The figure shows the relative amount of glycerate in the sum of the pool size of serine and glycerate in comparison to the error.

In summary, since all estimates shown here correspond to a comparable error, the behaviour of the reaction with respect to the reversibility, the value of the exchange flux, cannot be determine from the time-course data. In such cases the only the sum of pool size is determined.

2 SUPPLEMENTARY TABLES

Supplementary Table 1. Composition of the 5 elementary flux modes M that generate the steady-state net flux distributions, M^1 photorespiration and the synthesis of: M^2 starch, M^3 sucrose, M^4 trehalose, M^5 amino acids. All fluxes $F_{i,j}$ of a mode M denote a net flux from pool i to pool j. The indicies correspond to the one given in **Supplementary Figure 1**. Indented fluxes indicate an in- or out-flux from of a pool of a multi-molecular reaction. All fluxes are denoted in units of carbon atoms taking part in a reaction, such that the stoichiometric coefficient is factored in the flux value. The modes are normed such that the out-fluxes of the system, denoted by $*$ correspond to one carbon atom. In the current analysis the mode towards the synthesis of amino acids is constrained to zero.

**flux**	M^1	M^2	M^3	M^4	M^5
$F_{(0,CO_2),1}$	6	6	6	6	6
$F_{0,1}$	5	5	5	5	5
$F_{CO_2,1}$	1	1	1	1	1
$F_{(0,O_2),(1,15)}$	10	0	0	0	0
$F_{0,1}$	6	0	0	0	0
$F_{0,15}$	4	0	0	0	0
$F_{1,2}$	15	6	6	6	5
$F_{2,3}$	6	3	2	2	2
$F_{2,0}$	9	3	3	3	3
$F_{3,4}$	6	3	2	2	2
$F_{4,5}$	0	1	0	0	0
$F_{4,0}$	6	2	2	2	2
$F_{5,6}$	0	1	0	0	0
$F_{6,7}$	0	1	0	0	0
$F_{7,O}$	0	1	0	0	0
$F_{2,8}$	0	0	1	1	0
$F_{8,9}$	0	0	1	1	0
$F_{9,10}$	0	0	$\frac{1}{2}$	1	0
$F_{10,11}$	0	0	$\frac{1}{2}$	$\frac{1}{2}$	0
$F_{11,12}$	0	0	$\frac{1}{2}$	$\frac{1}{2}$	0
$F_{(9,12),13}$	0	0	1	0	0
$F_{9,13}$	0	0	$\frac{1}{2}$	0	0
$F_{12,13}$	0	0	$\frac{1}{2}$	0	0
$F_{13,*}$	0	0	1	0	0
$F_{(10,12),14}$	0	0	0	1	0
$F_{10,14}$	0	0	0	$\frac{1}{2}$	0
$F_{12,14}$	0	0	0	$\frac{1}{2}$	0
$F_{14,*}$	0	0	0	1	0
$F_{15,(16,CO_2)}$	4	0	0	0	0
$F_{15,*}$	1	0	0	0	0
$F_{15,16}$	3	0	0	0	0
$F_{16,17}$	3	0	0	0	0
$F_{17,1}$	3	0	0	0	0
$F_{1,18}$	0	0	0	0	1
$F_{18,*}$	0	0	0	0	1

Supplementary Table 2.Values of the parameters of the input models. The parameters used for the analysis, which describe the approximation of the generating fraction as $y_s^{\text{input}} = A_s e^{-a_s t} + B_s e^{-b_s t}$ where $A_s + B_s = 1$, for every s, $1 \leq s \leq 3$. The shown values are used for all scenarios.

	**parameter**			
	A	B	a (s^{-1})	b (s^{-1})
y_1^{input}	0.6956	0.3044	0.0076	0.0003
y_2^{input}	0.7615	0.2385	0.0133	0.0008
y_3^{input}	0.7690	0.2310	0.0200	0.0014

Supplementary Table 3. Parameters of the model and Optimization. The table shows the parameter of the model in the different scenarios. The minimization of the VWSS was done by the Subplex algorithm (Rowan, T. (1990). Functional Stability Analysis of Numerical Algorithms. PhD thesis, The University of Texas at Austin.) implemented as a part of the library NLopt version 2.3. in C programming language (http://ab-initio.mit.edx/nlopt). As stopping criteria a relative change for each parameter lower than 0.001 was used. The optimization is repeated for 100 times with different initial conditions and the best one is chosen. The initial conditions for each repetition are randomly generated from distribution shown in the table below, in which the uniform distribution with minimum a and maximum b is indicated by $\mathcal{U}(a, b)$ and normal distribution with mean μ and standard deviation σ by $\mathcal{N}(\mu, \sigma)$. In addition, the table lists the upper and lower boundaries of the parameters used for the optimization. The parameters ϕ_r^{ex} were used to express the exchange fluxes F_r^{ex} by: $F_r^{ex} = \gamma \frac{\phi_r^{ex}}{1-\phi_r^{ex}}$. This hyperbolic transformation allows an speed-up of the optimisation procedure, since the algorithm more easily switch from low to high exchange fluxes. The value of γ was set to 5, based on experience.

**parameter**	**number**	**initial distribution**	**boundaries**	**description**
Scenario A				
$\phi^{(n)}$	4	$\mathcal{U}(0, 20)$	$(0, 20)$	describe the steady-state net flux distribution
ϕ_r^{ex}	7	$\mathcal{U}(0, 0.6)$	$(0, 0.98)$	describe the exchange flux F_r^{ex} of reaction r
φ_m	13	$\mathcal{U}(0, 0.5)$	$(0, 0.8)$	inactive fraction of a metabolite m
c_n	18	-	-	compartmentalized content of of pool n
A_s, a_s, b_s	9	-	-	parameters describing the 3 input models y_s^{input}
Scenario B				
$\phi^{(n)}$	4	$\mathcal{U}(0, 20)$	$(0, 20)$	describe the steady-state net flux distribution
ϕ_r^{ex}	7	$\mathcal{U}(0, 0.6)$	$(0, 0.98)$	describe the exchange flux F_r^{ex} of reaction r
φ_m	13	$\mathcal{U}(0, 0.5)$	$(0, 0.8)$	inactive fraction of a metabolite m
c_n	18	$\mathcal{N}(\mu_{c_n}, \sigma_{c_n})$	$(\frac{1}{4}\sigma_{c_n}, 4\sigma_{c_n})$	compartmentalized content of pool n
A_s, a_s, b_s	9	-	-	parameters describing the 3 input models y_s^{input}
Scenario C				
$\phi^{(n)}$	4	$\mathcal{U}(0, 20)$	$(0, 20)$	describe the steady-state net flux distribution
ϕ_r^{ex}	5	$\mathcal{U}(0, .6)$	$(0, 0.998)$	describe the exchange flux F_r^{ex} of reaction r
φ_m	13	$\mathcal{U}(0, 0.5)$	$(0, 0.8)$	inactive fraction of a metabolite m
c_n	15	$\mathcal{U}(0.00001, 40000)$	$(0.00001, 40000)$	compartmentalized content of of pool n
A_s, a_s, b_s	9	-	-	parameters describing the 3 input models y_s^{input}

Supplementary Table 4. VWSS of the time-course per metabolite.

	scenario		
**metabolite**	**A**	**B**	**C**
3PGA	12.198	7.241	5.184
DHAP	22.766	11.493	6.406
FBP	13.021	12.379	14.370
F6P	2.982	3.129	1.600
G6P	5.454	4.419	4.225
G1P	5.661	4.205	3.558
ADPG	8.741	8.253	0.726
UDPG	7.732	8.313	5.247
Suc6P	18.191	15.158	2.595
Tre6P	8.807	8.725	8.756
Gly	6.683	6.559	1.438
Ser	5.785	5.052	4.395
Glyc	1.939	2.107	2.364
2PGA	6.478	5.381	3.274
sum	126.437	102.413	62.35

Supplementary Table 5. Estimates of exchange fluxes and compartmentalized content in Scenario C. The table lists best-fitting parameters. The column indicated by opt shows corresponding to the lowest observed error of 62.35. The additional columns show the lowest and the highest value of the parameters in the set of all obtained fit with an error lower than 63 and 65. For these estimations the upper limit for the exchange fluxes was 4995, the limits for the compartmentalized content was 0.00001 and 40000, respectively. In order to indicate these boundaries in the table the values shown are not normalized the the gross C fixation. Value in italic are constant.

**Exchange flux**	**opt**	**max error 63**		**max error 65**	
(nmol $gFW^{-1}s^{-1}$)		lowest	largest	lowest	largest
3PGA-DHAP	*4995*	*4995*	*4995*	*4995*	*4995*
$G6P_{pl} \leftrightarrow G1P_{pl}$	93.5046986741	0	2011.94231545	0	2785.17857143
$F6P_{cyt} \leftrightarrow G6P_{cyt}$	9.109944689	3.8498238	15.6446072157	0.9548550529	17.4233346189
$G6P_{cyt} \leftrightarrow G1P_{cyt}$	8.7452132295	4.7477097756	4995	4.7477097756	4995
$G1P_{cyt} \leftrightarrow$UDPG	1204.18984281	7.9309564511	4995	7.9309564511	4995
Ser$\leftrightarrow$Glyc	436.228379809	0	4995	0	4995
2PGA$\leftrightarrow$3PGA	*4995*	*4995*	*4995*	*4995*	*4995*
**Compartmentalized content**	**opt**	**max error 63**		**max error 65**	
(nmol gFW^{-1})		lowest	largest	lowest	largest
3PGA	*600.3*	*600.3*	*600.3*	*600.3*	*600.3*
DHAP	*47.64*	*47.64*	*47.64*	*47.64*	*47.64*
FBP_{pl}	0.189719	0.00001	1.415598	0.00001	36.407714
$F6P_{pl}$	1.273919	0.00001	8.875967	0.00001	16.058771
$G6P_{pl}$	0.00001	0.00001	21.457746	0.00001	282.429989
$G1P_{pl}$	42.246278	0.00001	85.815296	0.00001	143.305658
ADPG	0.00001	0.00001	0.079764	0.00001	0.244662
FBP_{cyt}	0.118764	0.00001	0.984638	0.00001	5.235068
$F6P_{cyt}$	4.609061	0.000037	26.974005	0.00001	49.016034
$G6P_{cyt}$	160.161578	0.000382	389.72484	0.00001	389.72484
$G1P_{cyt}$	118.535841	0.000199	614.996889	0.00001	614.996889
UDPG	1228.514373	330.213753	2131.42961	0.00001	2222.104271
Suc6P	2.742287	0.00001	21.399001	0.00001	24.580965
Tre6P	7614.955956	0.032728	20963.41225	0.032728	21154.732286
Gly	3002.201411	2385.080711	3598.089093	2208.484325	3610.254628
Ser	0.293212	0.00001	5444.846177	0.00001	5444.846177
Glyc	5348.075358	0.00001	6193.148685	0.00001	6375.310161
2PGA	*60.03*	*60.03*	*60.03*	*60.03*	*60.03*
Ser+Glyc	5348.36857	3746.343562	6211.13581	3336.107215	6388.278124

Supplementary Table 6. Estimates of inactive fractions. The table compares the estimated inactive fractions in Scenario A, B and C. They determine the size of the inactive and active pools (see **Supplementary Table 7**) from the metabolic content (see **Table 2**). The pools of the compartmentalized metabolites in the cytosol and chloroplast are indicated by the subscript cyt and pl. The optimal fits are denoted by opt. The lower and upper confidence limits (95%) are obtained by Monte-Carlo simulation.

	inactive fraction									
**Scenario**	**A**			**B**			**C**			
							**max error 63**		**max error 65**	
**Pool**	opt	lower	upper	opt	lower	upper	lowest	largest	lowest	largest
3PGA	0.03	0	0.08	0.04	0	0.08	0.063	0.073	0.062	0.075
DHAP	0.04	0.01	0.06	0.04	0.02	0.06	0.053	0.063	0.053	0.065
FBP_{pl}	0.25	0.2	0.33	0.25	0.18	0.31	0.252	0.255	0.246	0.256
$F6P_{pl}$	0.2	0.13	0.29	0.2	0.13	0.28	0.164	0.173	0.160	0.206
$G6P_{pl}$	0.25	0.19	0.31	0.24	0.19	0.31	0.232	0.240	0.232	0.248
$G1P_{pl}$	0.55	0.51	0.6	0.56	0.52	0.6	0.554	0.559	0.554	0.564
ADPG	0.02	0	0.06	0.03	0	0.08	0.042	0.044	0.028	0.045
FBP_{cyt}	0.25	0.2	0.33	0.25	0.18	0.31	0.252	0.255	0.246	0.256
$F6P_{cyt}$	0.2	0.13	0.29	0.2	0.13	0.28	0.165	0.173	0.160	0.206
$G6P_{cyt}$	0.25	0.19	0.31	0.24	0.19	0.31	0.232	0.240	0.232	0.248
$G1P_{cyt}$	0.55	0.51	0.6	0.56	0.52	0.6	0.554	0.559	0.554	0.564
UDPG	0.41	0.32	0.48	0.41	0.32	0.48	0.379	0.383	0.367	0.384
Suc6P	0.14	0.11	0.18	0.15	0.11	0.18	0.138	0.142	0.134	0.142
Tre6P	-	-	-	-	-	-	-	-	-	-
Gly	0.76	0.7	0.8	0.76	0.7	0.8	0.736	0.737	0.736	0.737
Ser	0.21	0.17	0.26	0.21	0.16	0.26	0.216	0.222	0.215	0.222
Glyc	0.27	0.19	0.35	0.28	0.19	0.35	0.287	0.292	0.287	0.293
2PGA	0.01	0	0.05	0.02	0	0.06	0.037	0.047	0.0368	0.049

Supplementary Table 7. Estimates of active pool sizes. The table compares the estimated active pool sizes in scenario A and B. The pools of the compartmentalized metabolites in the cytosol and chloroplast are indicated by the subscript cyt and pl. The active pool sizes is calculated from the metabolic contents (see **Table 2**) and the inactive fractions (see **Supplementary Table 6**). The optimal fits are denoted by opt. The lower and upper confidence limits (95%) are obtained by Monte-Carlo simulation. All values are denoted in nmol gFW^{-1} C atoms.

	**active pool size** (nmol gFW^{-1})					
**Scenario**	**A**			**B**		
**Pool**	opt	lower	upper	opt	lower	upper
3PGA	579.54	317.1	821.11	836.6	666.97	992.52
DHAP	45.8	42.23	49.55	45.84	42.26	49.89
FBP_{pl}	28.0	14.41	44.03	27.65	14.45	43.19
$F6P_{pl}$	140.37	91.76	189.25	136.43	89.99	177.82
$G6P_{pl}$	132.85	61.6	202.43	131.4	67.35	212.41
$G1P_{pl}$	2.5	1.58	3.49	2.54	1.69	3.4
ADPG	3.24	2.68	3.82	3.2	2.63	3.84
FBP_{cyt}	12.0	5.53	18.0	10.81	3.29	17.43
$F6P_{cyt}$	272.49	178.99	360.62	211.12	93.1	300.41
$G6P_{cyt}$	648.62	291.74	1018.95	755.09	350.06	1031.05
$G1P_{cyt}$	28.69	16.34	41.51	27.81	14.0	39.42
UDPG	127.61	88.98	172.41	129.34	90.63	177.08
Suc6P	8.43	2.13	15.26	6.68	2.09	14.87
Tre6P	1.92	0.95	2.94	1.92	0.9	2.96
Gly	259.89	192.17	350.25	263.97	185.5	353.42
Ser	10107.97	8462.8	11663.19	9489.76	7839.72	11035.06
Glyc	368.25	78.04	669.8	344.67	103.57	612.11
2PGA	59.18	32.95	81.43	61.92	38.79	88.28

3 SUPPLEMENTARY EQUATIONS

$$\text{3PGA}: \quad p_1 \frac{dx_1}{dt} = \frac{3}{5} F_{(O_2,0),(1,16)} y_3^{\text{input}} + \frac{1}{2} F_{(CO_2,0),1} y_3^{\text{input}} + F_{17,1}x_{17} + F_{2,1}x_2 + F_{18,1}x_{18} - (F_{1,2} + F_{1,18})\, x_1 \quad (1)$$

$$\text{DHAP}: \quad p_2 \frac{dx_2}{dt} = F_{1,2}x_1 - (F_{2,3} + F_{2,0} + F_{2,8} + B_{2,1})\, x_2 \quad (2)$$

$$\text{FBP}_{\text{pl.}}: \quad p_3 \frac{dx_3}{dt} = F_{2,3}\,(x_2)^2 - (F_{3,4} + F_{3,2})\, x_2 \quad (3)$$

$$\text{F6P}_{\text{pl.}}: \quad p_4 \frac{dx_4}{dt} = F_{3,4}x_3 - (F_{4,5} + F_{4,0})\, x_4 \quad (4)$$

$$\text{G6P}_{\text{pl.}}: \quad p_5 \frac{dx_5}{dt} = F_{4,5}x_4 + B_{6,5}x_6 - F_{5,6}x_5 \quad (5)$$

$$\text{G1P}_{\text{pl.}}: \quad p_6 \frac{dx_6}{dt} = F_{5,6}x_5 - (F_{6,7} + B_{6,5})\, x_6 \quad (6)$$

$$\text{ADPG}: \quad p_7 \frac{dx_7}{dt} = F_{6,7}x_6 - F_{7,0}x_7 \quad (7)$$

$$\text{FBP}_{\text{cyt.}}: \quad p_8 \frac{dx_8}{dt} = F_{2,8}\,(x_2)^2 + B_{9,8}x_9 - F_{8,9}x_8 \quad (8)$$

$$\text{F6P}_{\text{cyt.}}: \quad p_9 \frac{dx_9}{dt} = F_{8,9}x_8 + B_{10,9}x_{10} - (F_{9,10} + F_{9,13} + B_{9,8})\, x_9 \quad (9)$$

$$\text{G6P}_{\text{cyt.}}: \quad p_{10} \frac{dx_{10}}{dt} = F_{9,10}x_9 + B_{11,10}x_{11} \quad (10)$$

$$- (F_{10,11} + F_{10,14} + B_{10,9} + F_{10,15})\, x_{10} \quad (11)$$

$$\text{G1P}_{\text{cyt.}}: \quad p_{11} \frac{dx_{11}}{dt} = F_{10,11}x_{10} + B_{12,11}x_{12} - (F_{11,12} + B_{11,10})\, x_{11} \quad (12)$$

$$\text{UDPG}: \quad p_{12} \frac{dx_{12}}{dt} = F_{11,12}x_{11} - (F_{12,13} + F_{12,15} + B_{12,11})\, x_{11} \quad (13)$$

$$\text{Suc6P}: \quad p_{13} \frac{dx_{13}}{dt} = F_{(9,12),13}x_9x_{12} - F_{13,0}x_{13} \quad (14)$$

$$\text{Tre6P}: \quad p_{14} \frac{dx_{14}}{dt} = F_{(10,12),14}x_{10}x_{12} - F_{14,0}x_{14} \quad (15)$$

$$\text{Glycine}: \quad p_{15} \frac{dx_{15}}{dt} = \frac{2}{5} F_{(O_2,0),(1,15)} y_2^{\text{input}} - F_{15,(CO_2,16)}x_{15} \quad (16)$$

$$\text{Glycine}: \quad p_{15} \frac{dy_{15,1}}{dt} = \frac{2}{5} F_{(O_2,0),(1,15)} y_1^{\text{input}} - F_{15,(CO_2,16)}y_{15,1} \quad (17)$$

$$\text{Serine}: \quad p_{16} \frac{dx_{16}}{dt} = \frac{3}{4} F_{15,(CO_2,16)}\,(x_{15}y_{15,1}) + B_{17,16}x_{17} - F_{16,17}x_{16} \quad (18)$$

$$\text{Glycerate}: \quad p_{17} \frac{dx_{17}}{dt} = F_{16,17}x_{16} - (F_{17,1} + F_{17,16})\, x_{17} \quad (19)$$

$$\text{2PGA}: \quad p_{18} \frac{dx_{18}}{dt} = F_{1,18}x_1 - (F_{18,0} + B_{18,1})\, x_{18} \quad (20)$$

Note, the parameters p_i denoted the active pool size. The variable $y_{15,1}$ describes a necessary EMU-state-variable of mass-state zero for glycine. For further explanation and notation see: Heise et al (2014). Flux profiling of photosynthetic metabolism in intact plants. *Nat.Protoc.*9, 1803-1824
